# Judo for older adults: the coaches' knowledge and needs of education

**DOI:** 10.3389/fspor.2024.1375814

**Published:** 2024-04-02

**Authors:** Simone Ciaccioni, Flavia Guidotti, Federico Palumbo, Roberta Forte, Envic Galea, Attilio Sacripanti, Nuša Lampe, Špela Lampe, Toma Jelušić, Slaviŝa Bradić, Maria-Loredana Lascau, Alina Rodica-Borza, Raúl Camacho Pérez, Fernando Diéguez Rodríguez-Montero, Mesut Kapan, Kaya Gezeker, Laura Capranica, Antonio Tessitore

**Affiliations:** ^1^ Department of Movement, Human and Health Sciences, Italian University of Sport and Movement “Foro Italico”, Rome, Italy; ^2^ International Judo Federation Academy Foundation, Pembroke, Malta; ^3^ Judo Club Golovec, Ljubljana, Slovenia; ^4^ Zajednica Sportskih Udruga Grada Rijeke “Riječki Sportski Savez”, Rijeka, Croatia; ^5^ Judo Club Liberty Oradea, Oradea, Romania; ^6^ Club de Judo Newton, Madrid, Spain; ^7^ Izmir Alsancak Gymnastics Specialized Sports Club, Izmir, Türkiye

**Keywords:** judo, martial arts, older individuals, coaches, successful aging, education, survey, needs

## Abstract

This study aimed to explore the views of judo coaches on their perceived knowledge (PK) and needs for education (NE) for training older practitioners. In total, 470 international (Europe = 48%, Americas = 22%, Africa = 23%, Asia = 5% and Oceania = 2%) judo coaches (IJF: level 1 = 55,3%, level 2 = 33%; judo black belt: 3,4 ± 1,7 dan; F = 15%; university education: 68% >BA) responded an online survey encompassing demographic information and 35 items relevant to training older adults (Aging process; Safety and First Aid; Organization & Environment; Physiology and Fitness; Psychology & Mental Health; Teaching & Training) to be rated on a 7-point Likert scale for PK and NE. Non parametric statistics (*p* > 0.05) was applied to ascertain differences and relationships between PK and NE, respectively. A bivariate go-zone plot was used to highlight items with the lowest PK and the highest NE mean values. The coaches reported high PK (4.5 ± 0.3 pt) and NE (4.7 ± 0.1 pt) values, with significant higher PK values emerging for high education levels and judo experience. In considering their unique needs and special role, the judo coaches presented valuable insights to develop a sustainable educational curriculum tailored to train older judo practitioners.

## 1 Introduction

Sport federations mainly provide non-formal education to coaches for sustaining youth athlete development and enhancing their performance progress in specific sport disciplines. Conversely, coaches perceive a need of knowledge, capabilities and skills to train older individuals, who approach sports in later years (1–3). The dynamicity of this field is proved by a growing literature delving into the multifaceted nature of sport coaching, encompassing technical, tactical, psychological, pedagogical and andragogical domains spanning from recreational to elite levels across the lifespan (4–7).

Recently recognized and sustained by international policies and plans, sport coaching plays a strategic role in promoting healthy aging, given the increasing proportion of older adults in the world population and the growing need for active lifestyles (8–10). In providing guidance (e.g., sport-related and life skill development), support (e.g., motivation, team cohesion, long-term personal growth) and expertise (e.g., enhanced performance, professional feedback), coaches are instrumental in unlocking individuals' full potential and in pursuing success in sports and beyond across the life-span (11, 12). In particular, coaching-based programs provide personalized strategies to maximize the benefits of physical activity and optimize health outcomes in late adulthood (3, 13, 14). In fact, whilst active lifestyles have been strongly linked to fall prevention, enhanced functional abilities and improved well-being across behavioral, physiological, psychological, and social domains (15–22), the coaching-based sport interventions are essential resources to act effectively against age-related decline, chronic diseases risk, and mortality associated with sedentary behaviors (23–25). Hence, older adults could highly benefit from the involvement in structured multi-component physical activities (e.g., aerobic exercises, resistance training, and cognitive tasks) performed at least twice a week under the supervision of expert and competent coaches (26–30).

Several experimental studies (31–36) and systematic literature reviews (28, 37–41) emphasized the positive effects of judo training for the physical and mental health of older individuals. In fact, through its foundational principles of best use of energies and mutual welfare, judo proposes a variety of exercises (e.g., general gymnastics and calisthenics, standing-, breakfall- and ground techniques, specific choreographed and free movements), which could help maintain bone health, functional fitness (i.e., agility, coordination, endurance, flexibility, and strength), mental wellbeing (e.g., cognitive and psychological skills) and social connectedness with advancing age (28, 32, 42–46). Furthermore, judo practice could provide a supportive and inclusive environment for older practitioners, combating social isolation and promoting healthy aging (40–42). Whilst safe and sustainable sports programs should be based on unique requirements, capacities and abilities of sports professionals (40, 41, 47, 48), generally coaching certifications do not include information on the specific needs of older novice and expert practitioners (3, 49). Despite the growing research on judo training for older adults (7, 37, 40, 41), there is a paucity of studies focusing on the education of coaches in facilitating safe and effective training environments for this special population. To support coaches in effectively plan and conduct healthy judo training programs for older adults, in 2020 the European Commission has co-financed the “EDucating Judo Coaches for Older practitioners (EdJCO)” Project (622155-EPP-1-2020-1-IT-SPO-SCP) under the Erasmus + Sport Programme (3, 50). Thanks to a consortium of outstanding sports organizations (i.e., judo clubs from Croatia, Romania, Slovenia, Spain, and Türkiye) and educational institutions (i.e., University of Rome Foro Italico, Italy and International Judo Federation Academy—IJF—Foundation, Malta), the EdJCO project generated evidence- and eminence-based knowledge to develop a sound educational program for coaches aiming to train older judo practitioners (21, 51). Following a systematic literature review on the benefits and risks of judo training (40, 41) and the collection of experts' opinions regarding the most relevant knowledge on aging-related aspects coaches should possess (9, 13, 41), it is important to collect the opinion of potential end-users on their perceived knowledge and need of education in relation to different (e.g., bio-medical, psycho-physiological, and technical-organizational) dimensions of judo for older practitioners (52, 53). To engage a large number of international judo coaches in providing their valuable insights and perspectives, an online survey was deemed appropriate (51, 53).

Therefore, the aim of this eminence-based research was to investigate judo coaches' perceptions regarding their knowledge and need of education to develop and manage safe judo training for older individuals. In particular, the online survey methodology encompassed demographics information, and the following six key-domains identified through a systematic review, and international focus groups with 88 experts (21, 40, 41, 50): (i) aging process; (ii) safety and first aid; (iii) physiology and functional fitness; (iv) psychology and mental health; (v) organization and environment; and (vi) teaching and training. It was postulated that the primary results of the present work would yield pertinent and useful insights to enable guidelines for academic and judo organizations to develop effective educational programs tailored for coaches working with older individuals.

## 2 Materials and methods

Approved by the European Commission (622155-EPP-1-2020-1-IT-SPO-SCP) and the Institutional Review Board of the University of Rome Foro Italico (CAR 73/2021), the present study was performed under the Erasmus + Sport Collaborative Partnership “EdJCO—Educating Judo Coaches for Older practitioners”. By following high quality research guidelines for methodological procedures on data acquisition and analysis with ethical compliance conformed to the Declaration of Helsinki, this research guarantees stringent rigor, meaningful coherence and resonance of outcomes (14, 54, 55).

### 2.1 Study design

To develop an international educational program for judo coaches, incorporating educational modules and comprehensible content tailored to the specific needs of coaches from diverse social and cultural backgrounds, the present eminence-based phase of the EdJCO project applied an ethnographic research approach (56). To collect a broad range of opinions on key educational aspects of judo for older practitioners, the EdJCO research team agreed to purposefully apply the survey methodology for gaining access to international judo coaches (54). As a result of a systematic literature review (40, 41) and seven national focus groups (41), the research team identified six macro-areas representing the foundations of an edutional program for judo coaches of older practitioners (52): (i) Aging Process; (ii) Safety and First Aid; (iii) Organization and Environment; (iv) Physiology and Functional Fitness; (v) Psychology and Mental Health; and (vi) Teaching and Training. To gather coaches' opinions and insights about the macro-areas and their sub-domains, the survey methodology was considered as the most effective and appropriate method according to the following eight factors (51, 52, 54):

(1) Efficiency and reach. By distributing surveys electronically, researchers can efficiently reach and include a diverse, large and representative sample of coaches across different geographical locations in a short time;
(2) Anonymity and confidentiality. To foster open and genuine feedback, leading to more accurate insights into thoughts and evaluations, coaches may feel more comfortable providing honest and candid responses in a survey, as their identities remain anonymous;
(3) Standardization. Typically, surveys employ standardized questions, ensuring consistency in data collection, which allows for easy comparison and analysis of responses, and enables researchers to identify patterns and trends related to coaches' knowledge and training needs;
(4) Quantitative data. The statistical analysis of the data generated by means of surveys enables researchers to identify statistically significant trends and associations between variables, providing valuable insights into coaches' perceptions;
(5) Cost-effectiveness. Compared to data collection through in-depth interviews or focus groups, surveys are often more cost-effective, require fewer resources and can be administered to a larger number of participants, maximizing the return on investment;
(6) Time-efficiency for both researchers and participants. Coaches can complete surveys at their convenience, and data collection can be completed relatively quickly, allowing for a timely analysis of results;
(7) Structure and Focus. Surveys' structured approach allows researchers to obtain targeted information on coaches' perception of knowledge and training needs, since they are designed with specific research objectives in mind, ensuring that data collected aligns with the study's focus; and
(8) Replicability. The standardized nature of surveys makes them replicable, allowing other researchers to conduct similar studies and compare findings, contributing to the robustness of the research in the field of judo coaching.

To ensure accuracy and relevance of the outcomes, the EdJCO research team supported by the IJF Academy and the University of Rome “Foro Italico” scholars developed, translated and delivered the survey to judo coaches included in the IJF Academy and the Sport Bodies mailing lists.

### 2.2 Instrument and procedures of data collection

In the present study, the tailored online survey was developed to investigate coaches' perceptions regarding their perceived knowledge and needs of specific education for training older judo practitioners (Table 1). At the beginning of the survey, demographic questions were included to anonymously assess general (e.g., gender, age, nationality, highest educational attainment) and judo-specific (e.g., current judo level, judo-education level, experience as former athletes, coaching experience, weekly coaching volume, salary from judo-related activities, and specific experience of coaching older judo practitioners) information. Then, the survey encompassed a total 35 items grouped in 6 areas (Table 1) of perceived knowledge (PK)/need for education (NE) in relation to the coaching of older judo practitioners as follows:

Area 1—Aging Process: it focuses on understanding the effects of aging on the body organs and systems of older adults engaged in judo. Coaches in this area may need to be knowledgeable about age- and health-related changes and how to adapt training programs to accommodate older practitioners.
Area 2—Safety and First Aid: it centers on ensuring the safety and well-being of judo participants. Coaches need to be aware of a correct and appropriate hydratation and diet and well-versed in first aid practices, injury prevention, and emergency response procedures to create a safe training environment for older adults.
Area 3—Organization and Environment: it encompasses the effective setting and management of judo training sessions and events. Coaches should understand the importance of the social relationships connecting older adults and how to create a conducive learning environment and manage resources efficiently to enhance the coaching experience.
Area 4—Physiology and Functional Fitness: in this domain, coaches examine the physiological aspects of aging and functional fitness. Understanding the physical capabilities and limitations of older practitioners is essential to evaluate them and to tailor training regimens that optimize their performance and overall health.
Area 5—Psychology and Mental Health: it explores the psychological aspects of coaching older adults in judo. Coaches need to be sensitive to the mental and emotional well-being of their older athletes, fostering a supportive and positive training environment.
Area 6—Teaching and Training: coaches in this area should possess didactical and pedagogical skills and methods to deliver training programs that cater to the diverse needs and learning styles of older practitioners.

**Table 1:** Specific areas and related items on the specific knowledge (PK) and educational needs (NE) of coaches of older judo practitioners included in the survey of the present study.

Areas	Items (each evaluated in relation to the level of PK and NE)
Area 1	Aging Process
	1.1. Cardiovascular health
	1.2. Eyes health
	1.3. Hearing health
	1.4. Immune function
	1.5. Metabolic health
	1.6. Musculoskeletal health
	1.7. Healthy Sleep
Area 2	Safety and First Aid
	2.1. Diet and hydration
	2.2. Medical certificate and drug use
	2.3. Medical history
	2.4. Risk prevention
Area 3	Organization and Environment
	3.1. Economic status
	3.2. Family and social support
	3.3. Living conditions (e.g., autonomy and independence)
	3.4. Social relations and engagement
	3.5. Spaces (e.g., dojo, changing rooms, lighting etc.)
Area 4	Physiology and Fitness
	4.1. Evaluation
	4.2. Development and maintenance of functional fitness and physical capability
	4.3. Motor literacy knowledge
Area 5	Psychology and Mental Health
	5.1. Attitudes and motivations of older practitioners to practice judo
	5.2. Activation and relaxation status
	5.3. Body image
	5.4. Fear
	5.5. Individual and group empathy
	5.6. Mood and emotional status
	5.7. Psychological disorders
	5.8. Psychological trait and state
Area 6	Teaching and Training
	6.1. Adapted judo techniques for older practitioners (e.g., falling techniques in motion)
	6.2. Communication (e.g., efficacy and efficiency)
	6.3. Friendly context (e.g., openness, enjoyability)
	6.4. Group division or inclusion (e.g., age, athletic experience & capabilities, gender)
	6.5. Realistic goals through acute and long-term effects of judo training
	6.6. Training methodology and monitoring (e.g., workload, warmup & cooldown recovery time, rating of perceived exertion)
	6.7. Proactive participation and engagement (e.g., attendance)
	6.8. Variability of practice (e.g., introducing different sports to judo practice)

Each item was rated on a 7-pt Likert-type scale, where judo coaches had to specify their perceived PK (1 = little information; 7 = extensive information), and NE (1 = low need; 7 = very high need).

The judo coaches were asked to think about the needs of information to be included in an educational program for judo coaches of an older population of novice and expert judo practitioners and to rate their knowledge and needs of education. Each item was explained in depth by means of the PubMed definition or the respective terminology used in the National Language Medical controlled vocabulary thesaurus (S1). To avoid missing data, the questionnaire's design provided only close and compulsory answers.

### 2.3 Participants

To ensure a coaches' representativeness in relation to a variety of cultural and educational backgrounds, the recruitment strategy profited from the professional and structured network of the International Judo Federation Academy (>1,500 licensed coaches from 205 national judo federations). This approach guaranteed diversity in terms of geographical location, coaching experience, and organizational affiliation. A total of 470 respondents (32% response rate) filled in the on-line survey (Table 2), mostly males (85%), with a geographical representation spanning throughout five continents, and showing different ages, and highest educational attainment. Overall respondents were black-belt judoka (i.e., ranging from the 1st to the 8th dan), with a previous athletic experience at elite or competitive levels. The proportion of respondents reporting lower (≤3rd dan) and higher (4th–8th dan) levels of judo grading qualifications was 57% and 43%, respectively. As paid (55%) and unpaid (45%) coaches, participants reported judo-specific education spanning from national (12%) to international (88%) levels. Finally, a high coaching experience (>10 yrs; 68.9%) and a low (>3 h: 24%) or no (none: 46%) weekly coaching commitment with older adults emerged.

**Table 2:** Demographic characteristics of participants in the study.

Variables		Frequency of occurrence	
		(*n*)	(%)
Gender	Male	400	84.9
	Female	70	14.9
Age	<40 years	181	38.5
	40–49 years	151	32.1
	≥50 years	138	29.4
Geographical representation	Europe	227	48.3
	Americas	104	22.1
	Africa	106	22.6
	Asia	23	4.9
	Oceania	10	2.1
Highest academic attainment	≤EQF 5	149	31.7
	EQF 6	159	33.8
	≥EQF 7	162	34.5
Judo level	≤3rd dan	266	56.6
	≥4th dan	204	43.4
Former competition level	Elite	286	60.9
	Non-elite	184	39.0
Judo education level	International Level 1	260	55.3
	International Level 2	155	33.0
	National qualification	55	11.7
Coaching experience	<10 years	146	31.1
	10–20 years	151	32.1
	≥21 years	173	36.8
Coaching older judo practitioners	Yes	191	40.6
	No	279	59.4
Weekly coaching volume	None	216	46.0
	1–3 h	112	23.8
	4–6 h	78	16.6
	7–9 h	22	4.7
	10–13 h	17	3.6
	14–16 h	6	1.3
	17–19 h	6	1.3
	≥20 h	13	2.8
Salary for judo coaching	Paid	257	54.7
	Volunteer	213	45.3

EQF, european qualifications framework; *n*, sample size count.

### 2.4 Data analysis

Statistical analyses were conducted by means of the SPSS software (26.0; SPSS, Inc., Chicago, IL). Data were organized in relation to respondents' demographic characteristics, as displayed in Table 2. Furthermore, age, highest academic attainment, judo level, former competition level, judo education level, coaching experience, coaching older judo practitioners, salary for judo coaching were considered as independent variables for inferential statistics. In relation to the level of PK and NE, descriptive statistics (mean and standard deviation) were computed for individual items, for composite scores of items within each area, and overall scores for each PK and NE domains. To provide specific information of the perceived PK and NE, age (e.g., <40 years vs. 40–49 years vs. ≥50 years), highest academic attainment (e.g., ≤EQF 5 vs. EQF 6 vs. ≥EQF 7), judo level (e.g., ≤3rd dan vs. ≥4th dan), former competitive level (e.g., elite vs. non-elite), judo education level (e.g., IJF Level 1 vs. IJF 2 vs. National qualification), coaching experience (e.g., <10 years vs. 10–20 years vs. ≥21 years), coaching older judo practitioners (e.g., Yes vs. No), and salary for judo coaching (e.g., Paid vs. Volunteer) were considered as independent variables. A chi-square test was applied to verify potential between groups' unequal sample sizes in relation to the different independent variables. Then, the Kolmogorov-Smirnov test was applied to verify the normality of the distribution. The analysis was performed considering the following aspects: (i) effects of the selected independent variables on General PK, General NE, areas' PK, areas' NE, and individual items' PK and NE scores; (ii) correlations between dependent variables to explore the intertwined relationships between perceived PK and NE of both individual items and areas. Being data not normally distributed for all independent variables, a not parametric statistical approach was applied to evaluate differences (*p* ≤ 0.05) between the groups (e.g., 2-groups independent variables: Mann–Whitney *U* test; >2 groups independent variables: Kruskal-Wallis One-way ANOVA, and Mann–Whitney *U* test in case differences were observed in relation to the perceived PK and NE of individual items and areas. Furthermore, the Spearman's rank correlation coefficient with a cutoff value (≥0.7, *p* ≤ 0.01) was applied to verify relationships between dependent variables. Finally, to show the relationship between the perceived PK and NE of individual items, a bivariate go-zone plot was used.

## 3 Results

### 3.1 General, areas, and individual items K and NE scores

Results of the analysis in relation to the General, Areas, and individual items PK and NE scores is presented in the Supplementary Table S2. Regarding the General PK and NE scores, differences were observed in relation to judo level, former competition level, coaching experience, and involvement in coaching older judo practitioners. In particular, lower judo level respondents reported higher perceived General NE scores and lower General PK scores with respect to their higher-level counterparts (PK: ≤3rd dan = 4.5 ± 1.0 pt; ≥4th dan = 4.7 ± 0.9 pt; NE: ≤3rd dan = 4.8 ± 1.2 pt; ≥4th dan = 4.6 ± 1.2 pt.). A similar trend emerged for the coaching experience, with expert coaches showing the highest PK values and the lowest NE with respect to less experienced coaches (PK: <10 years = 4.6 ± 0.9 pt; ≥21 years = 4.8 ± 0.9 pt; NE: <10 years = 4.9 ± 1.2 pt; ≥21 years = 4.6 ± 1.2 pt), also in relation to the specific involvement in coaching older judo practitioners (PK: Yes = 4.8 ± 1.0 pt; No = 4.5 ± 1.0 pt; NE: Yes = 4.6 ± 1.3 pt; No = 4.8 ± 1.2 pt). Finally, former elite judoka showed higher PK scores with respect to their non-elite counterparts (PK: elite = 4.7 ± 0.9 pt; non-elite = 4.5 ± 1.0 pt).

Regarding the Areas and Individual items PK and NE scores, data are presented in relation to each independent variable. For age, differences emerged between the highest and oldest subgroups for several variables in relation to NE, with younger judoka presenting lower values with respect to their older counterparts. For the Areas, only Area 1 NE presented significant different scores (<40 years = 4.8 ± 1.6 pt; ≥50 years = 4.4 ± 1.3), which was confirmed by results in relation to the NE of items 1.3, 1.6, 1.7. Furthermore, differences emerged for the NE of items 3.1 (<40 years = 4.7 ± 1.5 pt; ≥50 years = 4.3 ± 1.4 pt), 3.2 (<40 years = 4.8 ± 1.5 pt; ≥50 years = 4.4 ± 1.4 pt), and 5.5 (<40 years = 5.0 ± 1.5 pt; ≥50 years = 4.6 ± 1.4 pt).

Regarding the respondents' education level, differences emerged for the highest academic attainment between ≤EQF 5 and ≥EQF 7 judoka, especially for PK of Areas 2 and 4, and individual items (1.1, 1.6, 2.1, 2.3, 2.4, 4.1, 4.2). In particular, participants with the lowest academic level showed lower (range: 3.9–4.6 pt) levels of PK with respect to their highest-level counterparts (range: 4.3–5.1 pt). Furthermore, a higher NE of ≤EQF 5 judoka emerged for item 1.2 with respect to their ≥ EQF 7 counterparts (≤EQF 5 = 4.3 ± 1.6 pt; ≥EQF 7 = 4.7 ± 1.5 pt). Conversely, the judo-related education level determined a difference only for item 6.7, with coaches holding the highest international qualification perceiving a higher level of PK (5.1 ± 1.3 pt) with respect to those having a different qualification (4.7 ± 1.4 pt).

For the other considered judo-specific independent variables, differences emerged for the participants' judo level in both the PK and NE of main Areas and individual items. For NE, ≤3rd dan judoka reported higher values (range: 4.9–5.0 pt) for Area 5, Area 6, and items 1.6, 5.2, 5.5–5.8, 6.1, and 6.3–6.8 with respect to ≥4th dan ones (range: 4.5–4.8 pt). For PK, lower-level judoka showed lower scores (range: 3.9–4.8 pt) for Areas 2, 5, and 6, and individual items 2.2, 2.4, 5.4, 6.1, 6.4, and 6.6, with respect to their higher-level counterparts (range: 4.2–5.0 pt). Regarding participants' former competitive level, differences emerged only for PK, with former elite judoka presenting higher scores (range: 4.2–5.0 pt) for Areas 2 and 4, and items 1.5, 1.6, 2.3, 2.4, 4.1, 4.2, 5.2, 6.4, 6.5, and 6.8 with respect to non-elite ones (range: 3.9–4.8 pt).

The judo coaching-related experiences determined the highest impact in participants' perceived level of PK and NE. In particular for PK, differences emerged for the Areas 1, 4, 5 and 6, and for the items 1.1–1.7, 4.1., 5.1–5.8, and 6.1–6.8, with a general trend of highly expert coaches displaying the highest scores (≥21 years range: 4.4–5.2 pt) with respect to their less experienced counterparts (<10 years and 10–20 years range: 4.0–5.1 pt, and 3.9–4.8 pt, respectively). Coherently, the opposite picture emerged for the NE domain of Areas 1 and 2, and items 1.1, 1.6, 2.1, 2.4, 5.7, 5.8, and 6.6 (≥21 years range: 4.5–4.7 pt; <10 years range: 4.9–5.2 pt; and 10–20 years range: 4.5–4.9 pt). Furthermore, coaches having experience with older judo practitioners showed higher values of PK for Areas 3, 5, and 6, and items 3.3, 3.4, 3.5, 5.1–5.7, and 6.1–6.8 (Yes range; 4.4–5.3 pt; No range: 4.1–4.9 pt), and lower levels of NE for Area 5, and items 5.2–5.4, 6.6, and 6.8 (Yes range; 4.5–4.7 pt; No range: 4.8–5.0 pt) with respect to those having no coaching experience with this special population. Finally, coaches receiving a salary showed higher levels of NE for Area 1 and 2, and items 1.2, 1.5, 1.7, and 2.1–2.4 with respect to their non-paid counterparts (Paid range: 4.7–4.9 pt; Volunteer range: 4.4–4.5 pt), whereas the opposite picture emerged for the PK of item 1.2 (Paid = 4.0 ± 1.5 pt; Volunteer = 4.4 ± 1.5 pt).

### 3.2 Correlations between dependent variables

Significant correlations between General PK and NE, and main Areas are presented in Table 3. Results reported coefficients ranging from 0.702 (General PK-Area2K) and 0.898 (General NE-Area 5 NE). Regarding PK, General PK was related with all the specific Areas' PK (CC range: 0.702–0.822). A similar picture emerged for NE (CC range: 0.792–0.898). Further relationships were shown also for NE of the Area 4 and 5, with significant coefficients emerging with the same domain of Areas 3.6. The correlations between the PK and NE of individual items are presented in the Supplementary Table S3. Intra-Areas significant correlations emerged between the majority of the items in relation to both PK and NE (PK—CC ranges: Area 1 = 0.722–0.888; Area 2 = 0.713–0.816; Area 3 = 0.732–0.833; Area 4 = 0.796–0.856; Area 5 = 0.701–0.869; Area 6 = 0.703–0.885; NE—CC ranges: Area 1 = 0.709–0.941; Area 2 = 0.729–0.871; Area 3 = 0.740–0.914; Area 4 = 0.901–0.934; Area 5 = 0.749–0.940; Area 6 = 0.834–0.950). For a graphical representation summary of the main effects see also Supplementary Figure S4.

**Table 3:** Correlation coefficients between general and Areas’ PK and NE domains.

Correlations—General K and NE; General areas’ K and NE								
Variables		General PK	Area1 NE	Area2 NE	Area3 NE	Area4 NE	Area5 NE	Area6 NE
General NE	CC		.792**	.786**	.837**	.834**	.898**	.866**
	p		>.001	>.001	>.001	>.001	>.001	>.001
Area1 PK	CC	.759**						
	p	>.001						
Area2 PK	CC	.702**						
	p	>.001						
Area3 PK	CC	.720**						
	p	>.001						
Area4 PK	CC	.746**						
	p	>.001						
Area4 NE	CC				.711**		.713**	.743**
	p				>.001		>.001	>.001
Area5 PK	CC	.820**						
	p	>.001						
Area5 NE	CC				.732**	.713**		.781**
	p				>.001	>.001		>.001
Area6 PK	CC	.822**						
	p	>.001						

PK, perceived knowledge; NE, need of education; CC, correlation coefficient.

** >.001.

### 3.3 Bivariate go-zones

**Figure 1:**
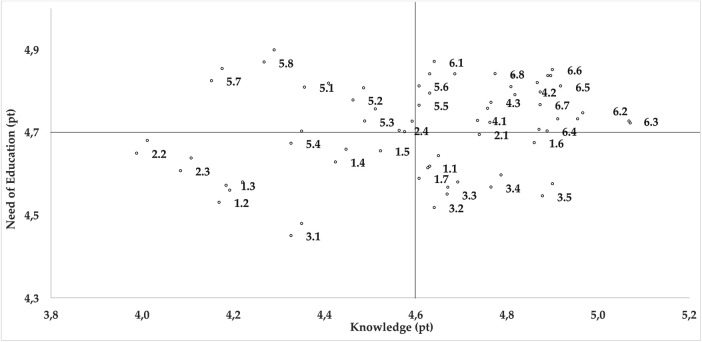
Go-zones in relation to the perceived knowledge (PK) and need of education (NE). The points (e.g., 5.7, 3.1) presented in this figure refer to the specific areas and related items of PK and NE as specified in Table 1.

Figure 1 represents the level of NE (y axis) and PK (x axis) of the mean scores of the items, plotted in four quadrants. In general, the coaches reported high PK (4.6 ± 0.3 pt) and NE (4.7 ± 0.1 pt) values. Based on participants' perceptions, the top left quadrant (I) showing high NE and low PK levels, includes five items (5.1–5.3, 5.7, 5.8); the top right quadrant (II) represents high levels of both perceived NE and PK and includes 13 items (2.1, 4.1–4.3, 5.5, 6.1–6.8); the bottom left quadrant (III) shows low levels of both NE and PK, and includes 7 items (1.2–1.4, 2.2, 2.3, 3.1, 5.4); and the bottom right quadrant (IV) represents high PK and low NE levels, including 8 items (1.1, 1.6, 1.7, 3.2–3.5, 5.6). Two items (1.5 and 2.4) presented the means of both PK and NE. Independently from the level of PK, almost all the items grouped in the high NE zones (quadrant I and II), whereas only four items plotted across the edge of the III quadrant.

## 4 Discussion

In trying to identify the relevant knowledge coaches need to possess for ensuring effective and safe judo programs for older adults (7, 21, 40, 41, 50), the present study provides a thorough understanding of the coaches' perceived PK and NE, thus addressing the increasing importance of judo for older adults targeting potential benefits of active aging (28, 37, 40, 41). In general, for both PK and NE respondents reported positive values (>4 pt out of a 7-pt scale).

### 4.1 Coaches' perceived knowledge and needs of education in general and specific areas related to the training of older judo practitioners

The present findings showed that several education-, sport career-, coaching experience-related factors influence the coaches' perceived general PK and NE for training older judo practitioners. The highest perceived general PK and the lower NE of respondents with the highest judo levels (≥4th dan) and longest coaching tenures (≥21 years) indicates that non-formal and informal judo education determine a deep understanding of judo techniques and principles, which could positively impact their ability to work with older adults (3). The importance of coaching education and practice in shaping coaches' expertise cannot be underestimated, as experienced coaches likely acquire valuable insights and skills over time, especially when having direct experience with the needs and challenges of older practitioners (12, 47, 48). However, in reporting a high perceived NE despite their high perceived PK, the coaches call for a sound educational program to train safely and effectively this special population. In sport, learning is a multifaceted process influenced by interactions with the environment, which can occur in formal settings leading to degrees (e.g., academic courses), non-formal organized paths leading to sport-specific qualifications (e.g., sport federation courses), and informal education occurring in an unorganized manner (e.g., personal experiences, peer learning, etc.) (1, 2, 57). Actually, non-formal and informal learning in sport offer several opportunities for personal growth, cooperation, and responsible behaviors (3, 11, 57). Moreover, in connecting people from diverse backgrounds, in promoting social understanding, and in breaking barriers, sport practice can be aligned with the principles of sustainable development (48, 58).

In the present study, the relatively lower NE displayed by older coaches (age ≥50 years) suggests that they might have added to their extensive coaching experience also personal experiences in age-related changes, which enables a more comprehensive understanding of appropriate training strategies addressing the needs of older adults (14). In fact, coaching experience emerged as a prominent factor influencing coaches' perceived PK and NE, since highly experienced coaches (≥21 years) displayed superior PK scores in aging process, physiological and psychological health and teaching and training aspects, highlighting the positive effect of accumulated experience on coaches' expertise (57). In particular, coaches declaring a direct experience with older judo practitioners exhibited higher PK scores in organizational and environmental aspects (e.g., older adults' living conditions, social relations and spaces-related information), highlighting the benefits of informal education to address relevant practicalities related to training (14). Conversely, the lower PK scores shown by coaches with the lowest academic level (≤EQF 5) emphasize the relevant role of formal education in enriching coaches' understanding of judo principles and appropriate training methods for older adults especially at an early stage of a coaching career (3, 40, 41). The significant highest NE scores for paid coaches regarding bio-medical aspects of aging (e.g., musculoskeletal system, healthy eyes and sleep) highlight that professional coaches exhibit high motivations and commitment, potentially influencing their perceived educational needs to be ready for engaging with individuals affected by age-related degenerative processes (47, 59). An apparent paradox could be the highest PK values for eyes health reported by volunteers. Despite special populations require to be trained by specialized personnel, volunteers might have a practical experience deriving from engaging with people with disabilities more often when compared to professional coaches (60).

These findings highlight the potential misconception that a high level of experience equates to comprehensive knowledge, not requiring further education. In fact, in the present study coaching experience plays a significant role in PK with respect to formal education. However, the coaches need for specific knowledge areas would support a valuable formal education. Although this study cannot definitively answer this question, it is important to underline the possibility of an overestimated PK due to extensive experience. Still acknowledging the importance of informal learning and the significance of non-formal education, it is also crucial to warn against the potential pitfalls of relying on experience when formal education may be necessary to acquire specialized knowledge (1, 2, 57).

### 4.2 Correlational analysis of coaches' perceived knowledge and educational needs regarding judo for older adults

The investigation of the interrelationships between the coaches' perceived PK and NE in the specific areas of judo for older practitioners allows a comprehensive, interdisciplinary approach to coaching education for better accommodating the training to the specific requirements of older adults (3). The significant intra-areas correlations between most items of both the PK and NE domains uncover a broader understanding that judo for older practitioners has to be based on a holistic approach to the individuals, with sound training plans and teaching methods taking into account the age, the psycho-physiological status, the fitness level, and the safety of the practitioners (1, 3, 13, 14, 42). Therefore, comprehensive educational programs encompassing both theoretical knowledge and practical exercises (e.g, falling techniques in motion, calisthenics, kata) are needed to optimize the coaches' capability to train older adults (21, 40, 41, 47).

### 4.3 Bivariate go zones on coaches' knowledge and educational needs

To provide a synthetic visual representation of the coaches' perceptions regarding their knowledge and educational needs in judo training for older adults, the bivariate go zones' plot divides the mean scores of individual items into four quadrants, each reflecting different combinations. Representing a group of factors where coaches perceive high NE but possess relatively low levels of PK, five aspects were included in Quadrant I: (i) Attitudes and motivations of older practitioners to practice judo, which could highly influence the practitioners’ adherence to the coaching program (35); (ii) Activation and relaxation status, which highlights the age-related physiological responses to training needing tailored strategies to optimize performance and well-being (13, 55); (iii) Body image, which could determine relevant concerns with advancing age and affect long-term engagement (32); (iv) Psychological disorders, which can be challenging to be recognized and addressed, although their early identification is crucial for providing appropriate support and intervention (61); (v) Psychological traits and states, which can aid coaches in tailoring approaches to enhance performance and overall mental well-being (61, 62). Therefore, targeted educational interventions and training programs are necessary to address these crucial areas to help judo coaches to be equipped to handle adequately older practitioners (47).

Despite high levels of PK, Quadrant II indicates aspects where coaches perceive high NE, therefore calling for further educational opportunities to deepen their expertise and to enhance their ability to meet effectively the specific demands of older judo practitioners (3). In particular, all the physiological and teaching and training-related aspects and some psychological factors (e.g., empathy, mood) appear in this quadrant, emphasizing the importance of tailored training, inclusivity, effective communication, goal setting, and practice diversification to promote athletes' well-being and progress. In fact, coaching judo for older individuals is a complex task requiring specialized and updated knowledge. Thus, coaches recognize the need of a lifelong education to deal with the specific challenges and nuances of training in light of the continuously evolving sports sciences, with research providing new insights into the training and coaching process. Indeed, since older judo practitioners may have different fitness levels, health conditions, and psychological traits, they necessitate a more individualized coaching approach and tailored training methods, ensuring an effective monitoring and workload management within a friendly and inclusive environment of practice where older individuals could feel welcomed, motivated, and supported throughout their judo journey (3, 40, 41). At the same time, coaches indicate their willingness to explore innovative approaches to improve their athletes' experience.

Quadrant III reports the relative low NE and PK levels in specific areas. Whilst coaches may not consider these factors requiring immediate concerns, continuous monitoring and evaluation of their coaching performance in these areas remain essential to ensure comprehensive support for older judo practitioners (54, 63). Finally, quadrant IV represents aspects where coaches possess high levels of PK, but they perceive relatively low NE. Since coaches already have here a strong understanding and consider their training needs minimal, they are well-equipped to handle the specific demands of older adults and may serve as resources for sharing their expertise with others (64).

In considering that most items fell within the high NE zones (quadrants I and II), it is possible to infer that coaches perceive a substantial need for additional education across multiple areas. This suggests that comprehensive coaching education programs should be designed to target these priority areas to bridge the gap between coaches’ perceived needs and their existing knowledge base (21, 40, 41). Additionally, the existence of items along the edge of quadrant III implies that there are a few aspects that coaches consider as low priority and for which they perceive a limited need for training. Whilst these areas may not be of immediate concerns, continuous assessment and development in all aspects of coaching remain crucial to ensure a well-rounded approach to working with older judo practitioners (63). Coaches' willingness to seek further education suggests their commitment to staying updated with the latest scientific findings and integrating evidence-based practices into their coaching methodologies.

These results present some convergences and discrepancies with literature of senior sport. Across sport disciplines, coaches presented a need of education to tailor training for optimizing sport performance and wellbeing of older athletes, and for providing them with an adequate psychological support (65–67). However, whilst judo coaches prioritize a holistic educational approach in line with the martial arts philosophies, the main focus of endurance sports coaches is on gradual adaptations to training volume and intensity to mitigate age-related injury risks (66), of strength-based sports coaches is on progressive resistance training to preserve muscle mass and strength with advancing age (65), and of team sports coaches is on cognitive agility and strategic adjustments to accommodate the age-related changes in physical capabilities (67). These comparisons enrich our understanding of coaching education for training older practitioners tailored to the unique demands of various sports contexts (9).

To gain a more comprehensive understanding of a tailored educational program for coaching judo for older individuals, the EdJCO research team have combined the survey with other data collection methods (7), namely a systematic literature review (40, 41) and seven national focus groups with experts (41). Carefully designed and complemented with other methods (54), the survey-based methodology provided valuable insights into judo coaches' thoughts and evaluations from a heterogeneous and large sample with the aim to significantly contribute to enhancing awareness among coaches, sports scientists, and sport bodies regarding judo training for older individuals. Moreover, in involving coaches from several countries, it is possible to hypothesize that this study captured different perspectives useful for understanding coaching practices in judo for older adults, thereby enhancing the generalizability of the findings. However, some potential limitations of survey-based methodologies need to be acknowledged. In particular, coaches' responses may be influenced by potential subjective bias always present in the qualitative research (68). Therefore, the lack of opportunity for in depth responses limited the further participants' elaboration of their thoughts. Thus, future research encompassing focus groups with judo coaches is needed to substantiate the present findings and to allow sound generalizations.

## 5 Conclusions

In conclusion, our study highlights the crucial role of coaches' knowledge and education needs in facilitating effective and safe judo programs for older adults. Coaches with higher judo levels, extensive experience, and involvement with older practitioners displayed greater general knowledge, emphasizing the value of expertise and practical experience. However, targeted educational programs are needed to address the specific training needs of coaches with lower levels and limited experience. Tailored training programs should consider coaches' academic qualifications and previous competition experiences. Practical experience with older judo practitioners in coaching education can enhance coaches' abilities to meet their unique needs. By addressing knowledge gaps and education needs, we can promote the benefits of judo for older adults, encouraging their active engagement in physical activity and improving their overall well-being. Future research should focus on designing targeted educational interventions and investigating the impact of specialized coaching education on the quality of judo programs for older adults. Finally, the bivariate go zones analysis provides valuable guidance for developing targeted educational interventions for judo coaches, enhancing their expertise and optimizing the delivery of judo programs, ultimately promoting the older adults’ quality of life (21, 22, 31, 44).

## Data Availability

The original contributions presented in the study are included in the article/**Supplementary Material**, further inquiries can be directed to the corresponding author.

## References

[1] Cushion C Nelson L Armour K Lyle J Jones R Sandford R Coach learning and development: a review of literature. Sports Coach UK. (2010) 2010:1–102.

[2] De Houwer J Barnes-Holmes D Moors A. What is learning? On the nature and merits of a functional definition of learning. Psychon Bull Rev. (2013) 20:631–42. 10.3758/s13423-013-0386-3 23359420

[3] Callary B Young B Rathwell S. Coaching Masters Athletes: Advancing Research and Practice in Adult Sport. Abingdon: Routledge (2021).

[4] Bentzen M Alexander D Bloom GA Kenttä G. What do we know about research on parasport coaches? A scoping review. Adapt Phys Activ Q. (2020) 38:109–37. 10.1123/apaq.2019-0147 33296870

[5] Fusco A Capranica L Palumbo F Mosci G Ciaccioni S Doupona M Dual career experiences of elite coaches enrolled at university level. PLoS One. (2023) 18:e0283009. 10.1371/journal.pone.0283009 37053185 PMC10101392

[6] Rothwell M Stone J Davids K. Exploring niche construction in sport coaching: an ecological dynamics analysis. Sports Coach Rev. (2023) 12:209–31. 10.1080/21640629.2021.1974692

[7] Ciaccioni S Guidotti F Palumbo F Forte R Galea E Sacripanti A Development of a sustainable educational programme for judo coaches of older practitioners: a transnational European partnership endeavor. Sustainability. (2024) 16:1115. 10.3390/su16031115

[8] European Commission. Directorate General for Education, Youth, Sport, and Culture. Strategic Plan 2020–2024. Bruxelles: European Commission (2020).

[9] Dionigi RA Eime R Young BW Callary B Rathwell S. Coaching older adults (aged 55+). In: Ives B Potrac P Gale L Nelson L, editors. Community Sport Coaching. Abingdon: Routledge (2021). p. 147–66.

[10] Moustakas L Lara-Bercial S North J Calvo G. Sport coaching systems in the European Union: state of the nations. Int J Sport Policy Pol. (2022) 14:93–110. 10.1080/19406940.2021.1987291

[11] Ives B Potrac P Gale L Nelson L. Community Sport Coaching: Policies and Practice. Abingdon: Routledge (2021).

[12] Guidotti F Demarie S Ciaccioni S Capranica L. Relevant sport management knowledge, competencies, and skills: an Umbrella review. Sustainability. (2023) 15:9515. 10.3390/su15129515

[13] Spirduso WW Francis KL Macrae PG. Physical Dimensions of Aging. 2nd ed. Champaign, IL: Human Kinetics (2005).

[14] Rose DJ. Physical Activity Instruction of Older Adults. 2nd ed. Champaign, IL: Human Kinetics (2019).

[15] Brug J Van Der Ploeg HP Loyen A Ahrens W Allais O Andersen LF Determinants of diet and physical activity (DEDIPAC): a summary of findings. Int J Behav Nutr Phys Act. (2017) 14:150. 10.1186/s12966-017-0609-5 29100542 PMC5670716

[16] Carlin A Perchoux C Puggina A Aleksovska K Buck C Burns C A life course examination of the physical environmental determinants of physical activity behaviour: a “Determinants of Diet and Physical Activity” (DEDIPAC) umbrella systematic literature review. PLoS One. (2017) 12:e0182083. 10.1371/journal.pone.0182083 28787023 PMC5546676

[17] Condello G Puggina A Aleksovska K Buck C Burns C Cardon G Behavioral determinants of physical activity across the life course: a “DEterminants of DIet and Physical ACtivity” (DEDIPAC) umbrella systematic literature review. Int J Behav Nutr Phys Act. (2017) 14:58. 10.1186/s12966-017-0510-2 28464958 PMC5414221

[18] Cortis C Puggina A Pesce C Aleksovska K Buck C Burns C Psychological determinants of physical activity across the life course: a “DEterminants of DIet and Physical ACtivity” (DEDIPAC) umbrella systematic literature review. PLoS One. (2017) 12:e0182709. 10.1371/journal.pone.0182709 28817676 PMC5560721

[19] Jaeschke L Steinbrecher A Luzak A Puggina A Aleksovska K Buck C Socio-cultural determinants of physical activity across the life course: a ‘Determinants of Diet and Physical Activity’ (DEDIPAC) umbrella systematic literature review. Int J Behav Nutr Phys Act. (2017) 14:173. 10.1186/s12966-017-0627-3 29262864 PMC5738775

[20] O’donoghue G Kennedy A Puggina A Aleksovska K Buck C Burns C Socio-economic determinants of physical activity across the life course: a” DEterminants of DIet and Physical ACtivity" (DEDIPAC) umbrella literature review. PLoS One. (2018) 13:e0190737. 10.1371/journal.pone.0190737 29351286 PMC5774703

[21] Ciaccioni S Palumbo F Forte R Galea E Kozsla T Sacripanti A Educating judo coaches for older practitioners. Arts Sci Judo. (2022) 2:63–6.

[22] Ciaccioni S Pesce C Forte R Presta V Di Baldassarre A Capranica L The interlink among age, functional fitness, and perception of health and quality of life: a mediation analysis. Int J Environ Res Public Health. (2022) 19:6850. 10.3390/ijerph19116850 35682433 PMC9180674

[23] Chastin SF Buck C Freiberger E Murphy M Brug J Cardon G Systematic literature review of determinants of sedentary behaviour in older adults: a DEDIPAC study. Int J Behav Nutr Phys Act. (2015) 12:127. 10.1186/s12966-015-0292-3 26437960 PMC4595239

[24] Oliveira JS Sherrington C Amorim AB Dario AB Tiedemann A. What is the effect of health coaching on physical activity participation in people aged 60 years and over? A systematic review of randomised controlled trials. Br J Sports Med. (2017) 51:1425–32. 10.1136/bjsports-2016-096943 28320732

[25] Zubala A Macgillivray S Frost H Kroll T Skelton DA Gavine A Promotion of physical activity interventions for community dwelling older adults: a systematic review of reviews. PLoS One. (2017) 12:e0180902. 10.1371/journal.pone.0180902 28700754 PMC5507305

[26] Swanenburg J De Bruin ED Stauffacher M Mulder T Uebelhart D. Effects of exercise and nutrition on postural balance and risk of falling in elderly people with decreased bone mineral density: randomized controlled trial pilot study. Clin Rehabil. (2007) 21:523–34. 10.1177/0269215507075206 17613583

[27] Muir-Hunter SW Wittwer JE. Dual-task testing to predict falls in community-dwelling older adults: a systematic review. Physiotherapy. (2016) 102:29–40. 10.1016/j.physio.2015.04.011 26390824

[28] Ciaccioni S Condello G Guidotti F Capranica L. Effects of judo training on bones: a systematic literature review. J Strength Cond Res. (2019b) 33:2882–96. 10.1519/JSC.0000000000002340 29239994

[29] Kim ACH Park SH Kim S Fontes-Comber A. Psychological and social outcomes of sport participation for older adults: a systematic review. Ageing Soc. (2020) 40:1529–49. 10.1017/S0144686X19000175

[30] World Health Organization. Global status report on physical activity 2022: web annex: global action plan on physical activity monitoring framework, indicators and data dictionary. Global Status Report on Physical Activity 2022: Web Annex: Global Action Plan on Physical Activity Monitoring Framework, Indicators and Data Dictionary. World Health Organization (2022). p. 1–26.

[31] Delcastillo-Andrés Ó Toronjo-Hornillo L Toronjo-Urquiza MT Cachón Zagalaz J Campos-Mesa MDC. Adapted utilitarian judo: the adaptation of a traditional martial art as a program for the improvement of the quality of life in older adult populations. Societies. (2018) 8:57. 10.3390/soc8030057

[32] Ciaccioni S Capranica L Forte R Chaabene H Pesce C Condello G. Effects of a judo training on functional fitness, anthropometric, and psychological variables in old novice practitioners. J Aging Phys Act. (2019) 27:831–42. 10.1123/japa.2018-0341 31034297

[33] Ciaccioni S Capranica L Forte R Pesce C Condello G. Effects of a 4-month judo program on gait performance in older adults. J Sports Med Phys Fitness. (2020) 60:685–92. 10.23736/S0022-4707.20.10446-8 32438784

[34] Arkkukangas M Bååthe KS Ekholm A Tonkonogi M. A 10-week judo-based exercise programme improves physical functions such as balance, strength and falling techniques in working age adults. BMC Public Health. (2021) 21:1–8. 10.1186/s12889-021-10775-z 33865349 PMC8052647

[35] Ciaccioni S Pesce C Capranica L Condello G. Effects of a judo training program on falling performance, fear of falling and exercise motivation in older novice judoka. Ido Mov Culture J Martial Arts Anthropol. (2021) 21:9–17. 10.14589/ido.21.3.2

[36] Jadczak AD Verma M Headland M Tucker G Visvanathan R. A judo-based exercise program to reduce falls and frailty risk in community-dwelling older adults: a feasibility study. J Frailty Aging. (2023) 13:1–9. 10.14283/jfa.2023.17 38305437

[37] Valdes-Badilla P Herrera-Valenzuela T Ramirez-Campillo R Aedo-Munoz E Baez-San Martin E Ojeda-Aravena A Effects of Olympic combat sports on older Adults’ health status: a systematic review. Int J Environ Res Public Health. (2021) 18:7381. 10.3390/ijerph18147381 34299833 PMC8303637

[38] Chan U Ayliffe L Visvanathan R Headland M Verma M Jadczak AD. Judo-based exercise programs to improve health outcomes in middle-aged and older adults with no judo experience: a scoping review. Geriatr Gerontol Int. (2023) 23:163–78. 10.1111/ggi.14553 36737880 PMC11503564

[39] Ciaccioni S Castro O Bahrami F Tomporowski PD Capranica L Biddle SJH Martial arts, combat sports, and mental health in adults: a systematic review. Psychol Sport Exerc. (2023) 70:102556. 10.1016/j.psychsport.2023.102556 37949383

[40] Palumbo F Ciaccioni S Guidotti F Forte R Sacripanti A Capranica L Risks and benefits of judo training for middle-aged and older people: a systematic review. Sports. (2023) 11:68. 10.3390/sports11030068 36976954 PMC10058523

[41] Palumbo F Ciaccioni S Guidotti F Forte R Galea E Sacripanti A Need of education for coaching judo for older adults: the EdJCO focus groups. Sports. (2023) 11(8):1–18. 10.3390/sports11080143 PMC10458867 37624123

[42] Kano J. Kodokan Judo: The Essential Guide to Judo by Its Founder Jigoro Kano. New York, NY, USA: Kodansha International (2013).

[43] Muiños M Ballesteros S. Sports can protect dynamic visual acuity from aging: a study with young and older judo and karate martial arts athletes. Atten Percept Psychophys. (2015) 77:2061–73. 10.3758/s13414-015-0901-x 25893472

[44] Sakuyama N Kamitani T Ikumi A Kida M Kaneshiro Y Akiyama K. Assessment of the efficacy and safety of a judo exercise program in improving the quality of life among elderly patients. J Rural Med. (2021) 16:229–35. 10.2185/jrm.2021-008 34707732 PMC8527618

[45] Kujach S Chroboczek M Jaworska J Sawicka A Smaruj M Winklewski P Judo training program improves brain and muscle function and elevates the peripheral BDNF concentration among the elderly. Sci Rep. (2022) 12:13900. 10.1038/s41598-022-17719-6 35974038 PMC9381784

[46] Odaka M Kagaya H Harada T Futada Y Yamaishi A Sasaki M. Effect of ukemi practice in judo on fear of falling and mobility skills in healthy older adults. J Phys Ther Sci. (2023) 35:146–50. 10.1589/jpts.35.146 36744201 PMC9889217

[47] European Commission. Expert Group on Skills and Human Resources Development in Sport. Guidelines Regarding the minimum Requirements in Skills and Competences for Coaches. Belgium: European Commission Brussels (2020).

[48] Guidotti F Demarie S Ciaccioni S Capranica L. Knowledge, competencies, and skills for a sustainable sport management growth: a systematic review. Sustainability. (2023) 15:7061. 10.3390/su15097061

[49] Federation IJ. (2021). *Congress 2021 report of the Education and Coaching Director—06.2021—ENG*. Available online at: https://www.ijf.org/ijf/documents/11 (accessed January 24, 2024).

[50] Edjco (2022). *EDucating Judo Coaches for Older practitioners*. Available online at: https://edjco.eu/ (accessed January 24, 2024).

[51] Braun V Clarke V Boulton E Davey L Mcevoy C. The online survey as a qualitative research tool. Int J Soc Res Methodol. (2021) 24:641–54. 10.1080/13645579.2020.1805550

[52] Tracy SJ. Qualitative quality: eight “big-tent” criteria for excellent qualitative research. Qual Inq. (2010) 16:837–51. 10.1177/1077800410383121

[53] Hooley T Wellens J Marriott J. What is Online Research?: Using the Internet for Social Science Research. London: Bloomsbury Academic (2012).

[54] Boateng GO Neilands TB Frongillo EA Melgar-Quinonez HR Young SL. Best practices for developing and validating scales for health, social, and behavioral research: a primer. Front Public Health. (2018) 6:149. 10.3389/fpubh.2018.00149 29942800 PMC6004510

[55] Biddle SJH Mutrie N Gorely T Faulkner G. Psychology of Physical Activity. New York: Routledge (2021).

[56] Genzuk M. A synthesis of ethnographic research. Occasional papers series. In: Center for Multilingual, Multicultural Research, editors. Center for Multilingual, Multicultural Research, Rossier School of Education. Los Angeles: University of Southern California (2003). p. 1–10.

[57] Walker LF Thomas R Driska AP. Informal and nonformal learning for sport coaches: a systematic review. Int J Sports Sci Coach. (2018) 13:694–707. 10.1177/1747954118791522

[58] Goals., U.N.S.D. (2021). *The Sustainable Development Agenda*. Available online at: https://www.un.org/sustainabledevelopment/development-agenda/ (accessed January 24, 2024).

[59] Mclean KN Mallett CJ. What motivates the motivators? An examination of sports coaches. Phys Educ Sport Pedagogy. (2012) 17:21–35. 10.1080/17408989.2010.535201

[60] Townsend R Peacham G. 10 Reflections on coaching policy and practice in community disability sport. In: Ives B Potrac P Gale L Nelson L, editors. Community Sport Coaching: Policies and Practice. Abingdon: Routledge (2021). p. 1–14.

[61] Schaie KW Willis SL. Handbook of the Psychology of Aging. London: Academic Press (2010).

[62] Kvaal K Ulstein I Nordhus IH Engedal K. The spielberger state-trait anxiety inventory (STAI): the state scale in detecting mental disorders in geriatric patients. Int J Geriatr Psychiatry. (2005) 20:629–34. 10.1002/gps.1330 16021666

[63] Athanasopoulou A Dopson S. A systematic review of executive coaching outcomes: is it the journey or the destination that matters the most? Leadersh Q. (2018) 29:70–88. 10.1016/j.leaqua.2017.11.004

[64] Lyle J Cushion C. Sport Coaching Concepts: A Framework for Coaching Practice. Abingdon: Taylor & Francis (2016).

[65] Del Vecchio L Reaburn P. Mixed methods strength training for the masters athlete a review. J Australian Strength Cond. (2013) 21:1–10.

[66] Lepers R Stapley PJ. Master athletes are extending the limits of human endurance. Front Physiol. (2016) 7:613. 10.3389/fphys.2016.00613 28018241 PMC5149541

[67] West S Naar JJ Son JS Liechty T. Promoting team sport participation among older women. J Park Recreat Admi. (2019) 37:33–50. 10.18666/JPRA-2019-9118

[68] Bergen N Labonté R. “Everything is perfect, and we have no problems”: detecting and limiting social desirability bias in qualitative research. Qual Health Res. (2020) 30:783–92. 10.1177/1049732319889354 31830860

