# Supplementary material for: Judo for older adults: the coaches' knowledge and needs of education
Source: Front Sports Act Living. 2024 Apr 2;6:1375814. doi: 10.3389/fspor.2024.1375814 (PMC11018922; doi:10.3389/fspor.2024.1375814)

Supplementary Material 1. Survey

# Section 1

EdJCO (Educating Judo Coaches for Older Practitioners) Survey - Preliminary Information on Judo Coaches

This survey is reserved to judo coaches.

Answering the following survey, you give your consent to use your anonimous responses to the University of Rome “Foro Italico” (as data controller and data process in the framework of the European project EDJCO -EDucating Judo Coaches for Older practitioners - Project ref. 622155-EPP-1-2020-1-IT-SPO-SCP).

We thank you very much for your time!

1. **Your sex**
   1. Male
   2. Female
   3. Rather not say
2. **Your age class (years) *drop-down list**
   1. 20-29
   2. 30-39
   3. 40-49
   4. 50-59
   5. 60-69
   6. >70
3. **Your Continent *drop-down list**
   1. Africa
   2. Americas
   3. Asia
   4. Europe
   5. Oceania
4. **Your judo level (black belt) *drop-down list**
   1. 1 dan
   2. 2 dan
   3. 3 dan
   4. 4 dan
   5. 5 dan
   6. 6 dan
   7. 7 dan
   8. 8 dan
   9. 9 dan
   10. 10 dan
5. **In your athletic career, have you been an elite judoka (e.g., national judo federation/international IJF competition level)?**
   1. Yes
   2. No
6. **Your IJF Academy Level *drop-down list**
   1. None
   2. Level 1 (Judo Instructor)
   3. Level 2 (Judo Coach)
7. **As a judo (both certified and not certified) coach, your experience in years is: *drop-down list**
   1. 1-5
   2. 6-10
   3. 10-15
   4. 16-20
   5. 21-25
   6. 26-30
   7. >31
8. **Have you ever trained judoka older than 60 years (including Master/Veteran competitors)?**
   1. Yes
   2. No
9. **Have you ever trained novice (e.g., white/yellow belt) judoka older than 60 years?**
   1. Yes
   2. No
10. **How many hours/week do you currently coach judo for older individuals? *drop-down list**
    1. None
    2. 1-3
    3. 4-6
    4. 7-9
    5. 10-13
    6. 14-16
    7. 17-19
    8. ≥20
11. **Do you receive an economic remuneration (e.g., salary) for your judo coaching activity?**
    1. Yes
    2. No
12. **Education: do you have an academic degree (e.g., Level 5, Bachelor, Master, PhD)?**
    1. Yes (Level 5)
    2. Yes (Bachelor)
    3. Yes (Master)
    4. Yes (PhD)
    5. Not at all
13. **If yes, your academic degree is in (if not, please write "No"): *open answer**

# Section 2

**EdJCO (Educating Judo Coaches for Older Practitioners) Survey**

**Please think about the needs of information to be included in an educational programme for judo coaches of an older population of novice and expert judoka. On the two 7-pt scales, specify your knowledge (1=little information; 7=extensive information), and needs of education (1=low need; 7=very high need), for the items included in 6 domains (1.Aging process, 2.Safety and First Aid, 3.Organisation & Environment, 4.Physiology and Fitness, 5.Psychology & Mental Health, 6.Teaching & Training).**

1. **Aging process**

The gradual irreversible changes in structure and function of an organism that occurs as a result of the passage of time.

**Items description**

1.1.Cardiovascular health refers to the HEART and the BLOOD VESSELS by which BLOOD is pumped and circulated through the body.

1.2.Eyes health refers to the organ of sight (i.e., organs made up of a three-layered roughly spherical structure specialized for receiving and responding to light).

1.3.Hearing health refers to the ability or act of sensing and transducing ACOUSTIC STIMULATION to the CENTRAL NERVOUS SYSTEM. It is also called audition.

1.4.Immune function refers to the body's defense mechanism against foreign organisms or substances and deviant native cells. It includes the humoral immune response and the cell-mediated response and consists of a complex of interrelated cellular, molecular, and genetic components.

1.5.Metabolic health refers to the chemical reactions in living organisms by which energy is provided for vital processes and activities and new material is assimilated.

1.6.Musculoskeletal health refers to the MUSCLES, bones (BONE AND BONES), and CARTILAGE of the body.

1.7.Healthy Sleep refers to the readily reversible suspension of sensorimotor interaction with the environment, usually associated with recumbency and immobility.

- Rate your knowledge/experience on the following items:

1.None; 2.Very poor; 3.Poor; 4.Average; 5.Good; 6.Very good; 7.Excellent

- Rate your need of education on the following items:

1.None; 2.Very low; 3.Low; 4.Average; 5.High; 6.Very high; 7.Maximum

- 1. Cardiovascular health (e.g., blood pressure, pacemaker, bypass, etc.)
  2. Eyes health
  3. Hearing health
  4. Immune function
  5. Metabolic health (e.g., diabetes, stomach ulcers, insulin pump, etc.)
  6. Musculoskeletal health (e.g., bone density, artificial joints, etc.)
  7. Healthy Sleep (e.g., quantity and quality)

# Section 3

**EdJCO (Educating Judo Coaches for Older Practitioners) Survey**

**Please think about the needs of information to be included in an educational programme for judo coaches of an older population of novice and expert judoka. On the two 7-pt scales, specify your knowledge/experience (1=none; 7=excellent), and needs of education (1=none; 7=maximum), for the items included in 6 domains (1.Aging process, 2.Safety and First Aid, 3.Organisation & Environment, 4.Physiology and Fitness, 5.Psychology & Mental Health, 6.Teaching & Training).**

1. **Safety and First Aid**

Safety is the freedom from exposure to danger and protection from the occurrence or risk of injury or loss. It suggests optimal precautions in the workplace, on the street, in the home, etc., and includes personal safety as well as the safety of property.

First Aid is the emergency care or treatment given to a person who suddenly becomes ill or injured before full medical services become available.

**Items description**

2.1. Diet and hydration refers to regular course of eating and drinking adopted by a person.

2.2. Medical certificate and drug use refers to the compliance with a set of standards defined by medical personnels and the utilization of drugs (e.g. medicines, therapeutic substances) reported in individual hospital studies, governmental agencies of public health (e.g., FDA) studies, marketing, or consumption, etc.

2.3. Medical history refers to information (e.g., injuries, actual diseases, etc.) gained by a physician by asking specific questions, either of the patient or of other people who know the person and can give suitable information, with the aim of obtaining information useful in formulating a diagnosis and providing medical care to the patient.

2.4. Risk prevention refers to efforts and designs to reduce the incidence of unexpected undesirable events in various environments and situations (e.g., past and present injuries, signs of drop out/burn out, etc).

- Rate your knowledge/experience on the following items:

1.None; 2.Very poor; 3.Poor; 4.Average; 5.Good; 6.Very good; 7.Excellent

- Rate your need of education on the following items:

1.None; 2.Very low; 3.Low; 4.Average; 5.High; 6.Very high; 7.Maximum

- 1. Diet and hydration
  2. Medical certificate and drug use
  3. Medical history (e.g., injuries, actual diseases, etc.)
  4. Risk prevention (e.g., past and present injuries, signs of drop out/burn out, etc.)

# Section 4

**EdJCO (Educating Judo Coaches for Older Practitioners) Survey**

**Please think about the needs of information to be included in an educational programme for judo coaches of an older population of novice and expert judoka. On the two 7-pt scales, specify your knowledge/experience (1=none; 7=excellent), and needs of education (1=none; 7=maximum), for the items included in 6 domains (1.Aging process, 2.Safety and First Aid, 3.Organisation & Environment, 4.Physiology and Fitness, 5.Psychology & Mental Health, 6.Teaching & Training).**

1. **Organisation & Environment**

Organisation is groups of people working together in a structured manner to pursue common goals and objectives.

Environment is the external elements and conditions which surround, influence, and affect the life and development of an organism or population.

**Items description**

3.1. Economic status refers to personal or family variables such as household income, assets such as inherited wealth, savings, employment benefits, or ownership of homes or other property.

3.2. Family and social support refers to aid systems that provide assistance and encouragement to individuals with or without physical or emotional disabilities in order that they may better cope. Informal social support is usually provided by friends, relatives, or peers, while formal assistance such as COMMUNITY SUPPORT is provided by churches, groups, etc.

3.3. Living conditions refer to the state of quality of life as it exists or in flux (e.g., autonomy and independence).

3.4. Social relations and engagement refer to the reciprocal interaction of two or more persons and the involvement in community activities or programs.

3.5. Spaces refer to boundaries surrounding the individual's body which are maintained in relation to others (e.g., dojo, changing rooms, lighting etc).

- Rate your knowledge/experience on the following items:

1.None; 2.Very poor; 3.Poor; 4.Average; 5.Good; 6.Very good; 7.Excellent

- Rate your need of education on the following items:

1.None; 2.Very low; 3.Low; 4.Average; 5.High; 6.Very high; 7.Maximum

- 1. Economic status
  2. Family and social support
  3. Living conditions (e.g., autonomy and independence)
  4. Social relations and engagement
  5. Spaces (e.g., dojo, changing rooms, lighting etc.)

# Section 5

**EdJCO (Educating Judo Coaches for Older Practitioners) Survey**

**Please think about the needs of information to be included in an educational programme for judo coaches of an older population of novice and expert judoka. On the two 7-pt scales, specify your knowledge/experience (1=none; 7=excellent), and needs of education (1=none; 7=maximum), for the items included in 6 domains (1.Aging process, 2.Safety and First Aid, 3.Organisation & Environment, 4.Physiology and Fitness, 5.Psychology & Mental Health, 6.Teaching & Training).**

1. **Physiology and Fitness**

Physiology is the biological science concerned with the life-supporting properties, functions, and processes of living organisms or their parts.

Fitness, specifically physical fitness, is the ability to carry out daily tasks and perform physical activities in a highly functional state, often as a result of physical conditioning.

**Items description**

4.1. Evaluation (e.g., physical and technical tests and well-being questionnaires before, during and after training) refers to studies designed to assess the efficacy of programs. They may include the evaluation of cost-effectiveness, the extent to which objectives are met, or impact.

4.2. Development and maintenance of functional fitness and physical capability (e.g., aerobic capacity, coordination, flexibility, reactivity, strength, velocity of movement) refer to the physical capacity of the individual to meet ordinary and unexpected demands of daily life safely and effectively.

4.3. Motor (i.e., movement) literacy knowledge refers to the the ability to plan and execute basic motor skills (e.g., run, jump, catch, kick and throw) with agility, balance and co-ordination. It is connected to physical education, sport and sedentary behaviours.

- Rate your knowledge/experience on the following items:

1.None; 2.Very poor; 3.Poor; 4.Average; 5.Good; 6.Very good; 7.Excellent

- Rate your need of education on the following items:

1.None; 2.Very low; 3.Low; 4.Average; 5.High; 6.Very high; 7.Maximum

- 1. Evaluation (e.g., physical and technical tests and well-being questionnaires before, during and after training)
  2. Development and maintenance of functional fitness and physical capability (e.g., aerobic capacity, coordination, flexibility, reactivity, strength, velocity of movement)
  3. Motor literacy knowledge (e.g., physical education, sport and sedentary behaviours)

# Section 6

**EdJCO (Educating Judo Coaches for Older Practitioners) Survey**

**Please think about the needs of information to be included in an educational programme for judo coaches of an older population of novice and expert judoka. On the two 7-pt scales, specify your knowledge/experience (1=none; 7=excellent), and needs of education (1=none; 7=maximum), for the items included in 6 domains (1.Aging process, 2.Safety and First Aid, 3.Organisation & Environment, 4.Physiology and Fitness, 5.Psychology & Mental Health, 6.Teaching & Training).**

1. **Psychology & Mental Health**

Psychology is the science dealing with the study of mental processes and behavior in man and animals.

Mental health is defined as emotional, psychological, and social well-being of an individual or group.

**Items description**

5.1. Attitudes of older practitioners to practice judo refer to an enduring, learned predisposition to behave in a consistent way toward a given class of objects, or a persistent mental and/or neural state of readiness to react to a certain class of objects, not as they are but as they are conceived to be. Motivations refer to those factors which cause an organism to behave or act in either a goal-seeking or satisfying manner (e.g., to have fun). They may be influenced by physiological drives or by external stimuli.

5.2. Activation and relaxation status refers to the activity of vigilance/readiness of tone (presumed to be in response to sensory stimulation) or that reduces the feelings of tension and the effects of STRESS.

5.3. Body image refers to the individuals' concept of their own bodies.

5.4. Fear refers to the affective response to an actual current external danger which subsides with the elimination of the threatening condition (e.g., fear of contact with partners, falling, training barefoot, ground fighting, re-training)

5.5. Individual and group empathy refers to an individual/group's objective and insightful awareness of the feelings and behavior of another person/group. It should be distinguished from sympathy, which is usually nonobjective and noncritical. It includes caring, which is the demonstration of an awareness of and a concern for the good of others.

5.6. Mood and emotional status refers to those affective states which can be experienced and have arousing and motivational properties. Mood is connected to the feeling-tone accompaniment of an idea or mental representation.

5.7. Psychological disorders refer to psychiatric illness or diseases manifested by breakdowns in the adaptational process expressed primarily as abnormalities of thought, feeling, and behavior producing either distress or impairment of function (e.g., bipolarity, dementia).

5.8. Psychological trait is an habitual pattern of behaviour, thought, and emotion, whereas psychological state is a more transitory disposition.

- Rate your knowledge/experience on the following items:

1.None; 2.Very poor; 3.Poor; 4.Average; 5.Good; 6.Very good; 7.Excellent

- Rate your need of education on the following items:

1.None; 2.Very low; 3.Low; 4.Average; 5.High; 6.Very high; 7.Maximum

- 1. Attitudes and motivations of older practitioners to practice judo (e.g., to have fun)
  2. Activation and relaxation status
  3. Body image
  4. Fear (e.g., contact with partners, falling, training barefoot, ground fighting, re-training)
  5. Individual and group empathy
  6. Mood and emotional status
  7. Psychological disorders (e.g., bipolarity, dementia)
  8. Psychological trait and state (e.g., self-confidence)

# Section 7

**EdJCO (Educating Judo Coaches for Older Practitioners) Survey**

**Please think about the needs of information to be included in an educational programme for judo coaches of an older population of novice and expert judoka. On the two 7-pt scales, specify your knowledge/experience (1=none; 7=excellent), and needs of education (1=none; 7=maximum), for the items included in 6 domains (1.Aging process, 2.Safety and First Aid, 3.Organisation & Environment, 4.Physiology and Fitness, 5.Psychology & Mental Health, 6.Teaching & Training).**

1. **Teaching & Training**

Teaching is a formal and organized process of transmitting knowledge to a person or group.

Training is instructional programs in the care and development of the body.

**Items description**

6.1. Adapted judo techniques for older practitioners refer to judo-specif abilities to carry out a movement with determined results (e.g., falling techniques)

6.2. Communication refers to the exchange or transmission of ideas, attitudes, or beliefs between individuals or groups.

6.3. Friendly context (e.g., openness, enjoyability) refers to the surroundings, circumstances, environment, background or settings that determine, specify, or clarify the meaning of an event or other occurrence.

6.4. Group division or inclusion refer to the organisation of the judo group (e.g., age, athletic experience & capabilities, gender).

6.5. Realistic goals through acute and long-term effects of judo training refer to truthful end-result or objective, which may be specified or required in advance to be achieved through the immediate or durable practice of judo.

6.6. Training methodology and monitoring (e.g., workload, warmup & cooldown recovery time, rating of perceived exertion) refer to a series of steps (i.e., techniques, procedures, and programs) taken in order to conduct and evaluate the practice of judo.

6.7. Proactive participation and engagement (e.g., attendance) refer to a dynamic involvement in activities or programs of judo.

6.8. Variability of practice (e.g., introducing different sports to judo practice) refers to the inherent functional features of the neuromuscular system, which need functional changes

- Rate your knowledge/experience on the following items:

1.None; 2.Very poor; 3.Poor; 4.Average; 5.Good; 6.Very good; 7.Excellent

- Rate your need of education on the following items:

1.None; 2.Very low; 3.Low; 4.Average; 5.High; 6.Very high; 7.Maximum

- 1. Adapted judo techniques for older practitioners (e.g., falling techniques)
  2. Communication (e.g., efficacy and efficiency)
  3. Friendly context (e.g., openness, enjoyability)
  4. Group division or inclusion (e.g., age, athletic experience & capabilities, gender)
  5. Realistic goals through acute and long-term effects of judo training
  6. Training methodology and monitoring (e.g., workload, warmup & cooldown recovery time, rating of perceived exertion)
  7. Proactive participation and engagement (e.g., attendance)
  8. Variability of practice (e.g., introducing different sports to judo practice)

**Example:**


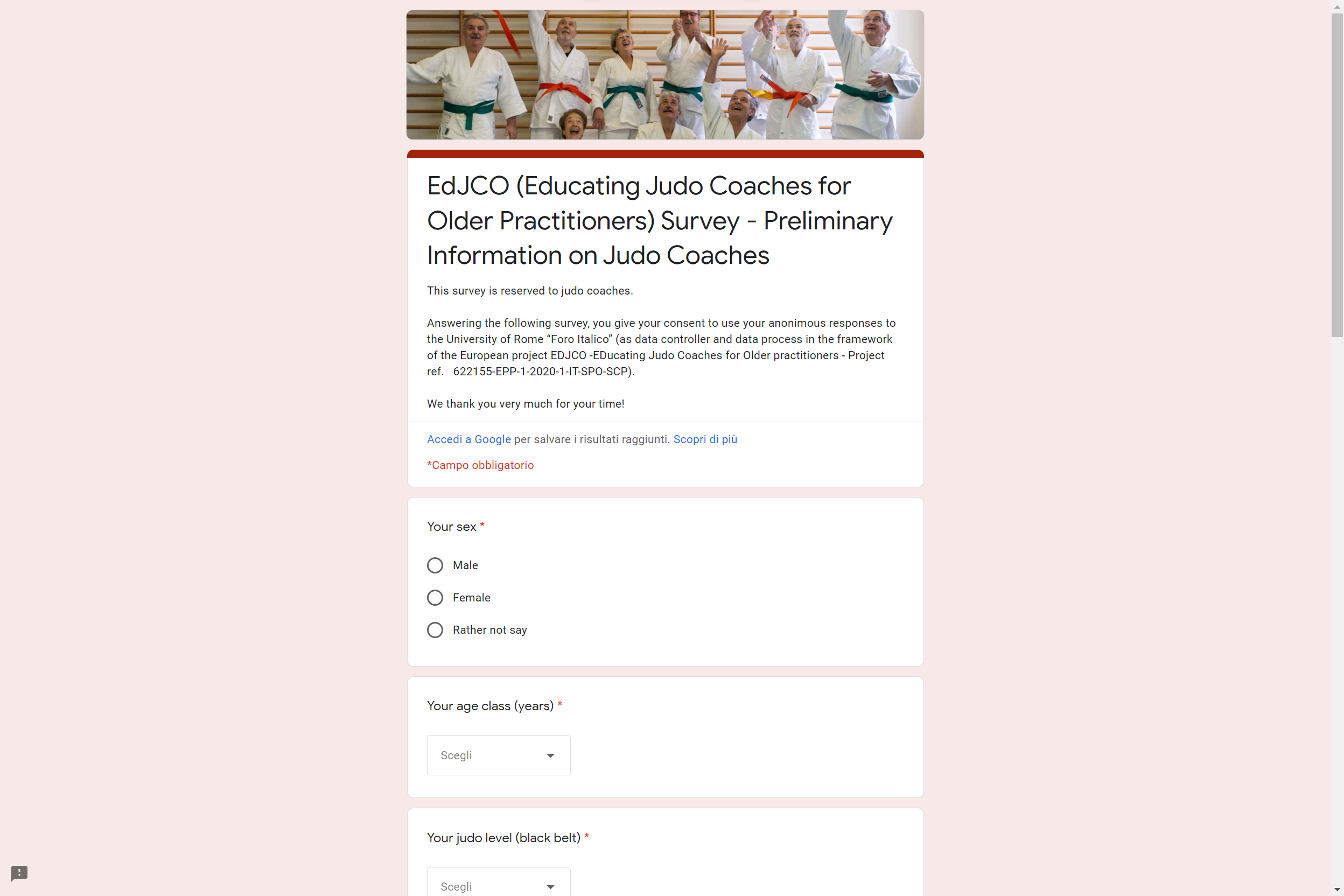


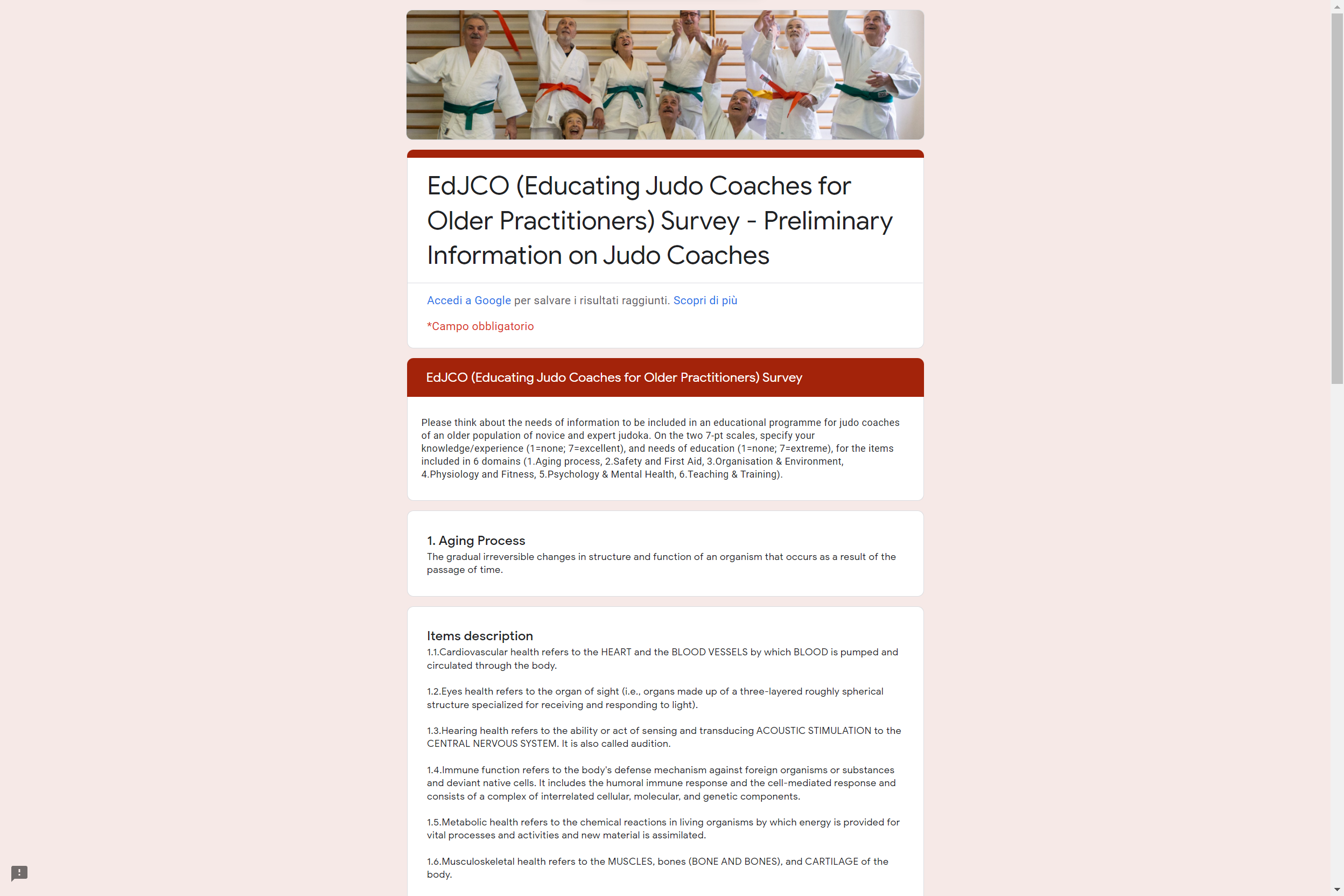


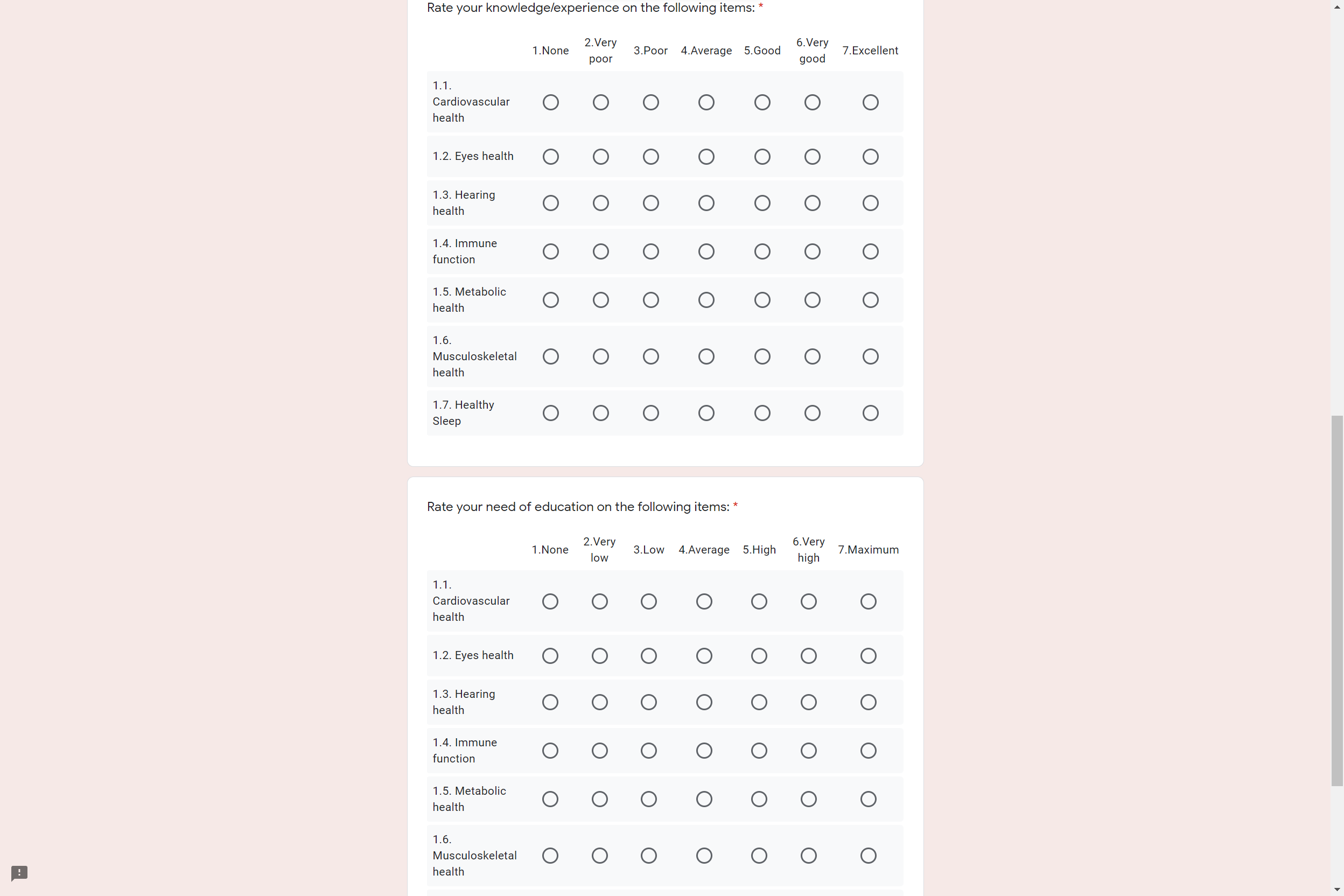

Supplement: Supplementary file 1 [file Datasheet1.docx]
