# Supplementary material for: Judo for older adults: the coaches' knowledge and needs of education
Source: Front Sports Act Living. 2024 Apr 2;6:1375814. doi: 10.3389/fspor.2024.1375814 (PMC11018922; doi:10.3389/fspor.2024.1375814)
Supplement: Supplementary file 2 [file Datasheet2.docx]

Supplementary Material 2. Scores

Mean and standard deviation of recorded scores (pt.) in relation to perceived Knowledge (PK) and Need of Education (NE). Differences are significant at p≤0.05.

|  |  |  |  |  |  | **Age** | | | | | | | | |  |
| --- | --- | --- | --- | --- | --- | --- | --- | --- | --- | --- | --- | --- | --- | --- | --- |
| **Variables** |  | **Overall** | | |  | **<40 years** | | | **40-49 years** | | | **≥50 years** | | |  |
| **General** | PK | 4.6 | ± | 1.0 |  | 4.7 | ± | 1.0 | 4.6 | ± | 1.0 | 4.6 | ± | 1.0 |  |
|  | NE | 4.7 | ± | 1.2 |  | 4.8 | ± | 1.3 | 4.7 | ± | 1.2 | 4.6 | ± | 1.2 |  |
| **General area** | Area1_PK | 4.5 | ± | 1.4 |  | 4.5 | ± | 1.4 | 4.4 | ± | 1.4 | 4.6 | ± | 1.4 |  |
|  | Area1_NE | 4.6 | ± | 1.5 |  | 4.8 | ± | 1.6 | 4.6 | ± | 1.4 | 4.4 | ± | 1.3 | * |
|  | Area2_PK | 4.4 | ± | 1.3 |  | 4.4 | ± | 1.3 | 4.3 | ± | 1.2 | 4.4 | ± | 1.2 |  |
|  | Area2_NE | 4.7 | ± | 1.4 |  | 4.8 | ± | 1.5 | 4.7 | ± | 1.4 | 4.6 | ± | 1.3 |  |
|  | Area3_PK | 4.7 | ± | 1.1 |  | 4.7 | ± | 1.1 | 4.7 | ± | 1.2 | 4.6 | ± | 1.1 |  |
|  | Area3_NE | 4.6 | ± | 1.4 |  | 4.7 | ± | 1.4 | 4.5 | ± | 1.4 | 4.4 | ± | 1.3 |  |
|  | Area4_PK | 4.8 | ± | 1.2 |  | 4.9 | ± | 1.2 | 4.8 | ± | 1.1 | 4.7 | ± | 1.2 |  |
|  | Area4_NE | 4.8 | ± | 1.5 |  | 4.8 | ± | 1.5 | 4.8 | ± | 1.5 | 4.7 | ± | 1.4 |  |
|  | Area5_PK | 4.4 | ± | 1.2 |  | 4.5 | ± | 1.2 | 4.5 | ± | 1.2 | 4.3 | ± | 1.2 |  |
|  | Area5_NE | 4.8 | ± | 1.4 |  | 4.9 | ± | 1.4 | 4.8 | ± | 1.4 | 4.7 | ± | 1.2 |  |
|  | Area6_PK | 4.9 | ± | 1.2 |  | 5.0 | ± | 1.3 | 4.8 | ± | 1.2 | 4.9 | ± | 1.2 |  |
|  | Area6_NE | 4.8 | ± | 1.5 |  | 4.9 | ± | 1.5 | 4.8 | ± | 1.5 | 4.6 | ± | 1.5 |  |
| **Area 1** | PK_1-1 | 4.7 | ± | 1.5 |  | 4.6 | ± | 1.5 | 4.6 | ± | 1.5 | 4.7 | ± | 1.5 |  |
|  | PK_1-2 | 4.2 | ± | 1.5 |  | 4.2 | ± | 1.6 | 4.1 | ± | 1.5 | 4.3 | ± | 1.4 |  |
|  | PK_1-3 | 4.2 | ± | 1.6 |  | 4.2 | ± | 1.6 | 4.2 | ± | 1.6 | 4.3 | ± | 1.5 |  |
|  | PK_1-4 | 4.4 | ± | 1.6 |  | 4.5 | ± | 1.6 | 4.4 | ± | 1.5 | 4.5 | ± | 1.5 |  |
|  | PK_1-5 | 4.6 | ± | 1.5 |  | 4.7 | ± | 1.5 | 4.5 | ± | 1.5 | 4.6 | ± | 1.5 |  |
|  | PK_1-6 | 4.9 | ± | 1.5 |  | 4.9 | ± | 1.5 | 4.8 | ± | 1.4 | 4.9 | ± | 1.5 |  |
|  | PK_1-7 | 4.6 | ± | 1.5 |  | 4.7 | ± | 1.6 | 4.5 | ± | 1.5 | 4.7 | ± | 1.5 |  |
|  | NE_1-1 | 4.6 | ± | 1.6 |  | 4.8 | ± | 1.6 | 4.6 | ± | 1.6 | 4.5 | ± | 1.5 |  |
|  | NE_1-2 | 4.6 | ± | 1.6 |  | 4.7 | ± | 1.7 | 4.5 | ± | 1.6 | 4.4 | ± | 1.4 |  |
|  | NE_1-3 | 4.6 | ± | 1.6 |  | 4.8 | ± | 1.7 | 4.5 | ± | 1.6 | 4.3 | ± | 1.4 | * |
|  | NE_1-4 | 4.7 | ± | 1.6 |  | 4.8 | ± | 1.7 | 4.6 | ± | 1.6 | 4.5 | ± | 1.4 |  |
|  | NE_1-5 | 4.7 | ± | 1.6 |  | 4.8 | ± | 1.7 | 4.7 | ± | 1.6 | 4.5 | ± | 1.4 |  |
|  | NE_1-6 | 4.7 | ± | 1.6 |  | 4.9 | ± | 1.7 | 4.7 | ± | 1.6 | 4.4 | ± | 1.6 | * |
|  | NE_1-7 | 4.6 | ± | 1.6 |  | 4.9 | ± | 1.7 | 4.6 | ± | 1.5 | 4.4 | ± | 1.4 | * |
| **Area 2** | PK_2-1 | 4.8 | ± | 1.3 |  | 4.8 | ± | 1.3 | 4.7 | ± | 1.2 | 4.8 | ± | 1.2 |  |
|  | PK_2-2 | 4.0 | ± | 1.5 |  | 3.9 | ± | 1.6 | 4.0 | ± | 1.5 | 4.1 | ± | 1.4 |  |
|  | PK_2-3 | 4.1 | ± | 1.5 |  | 4.1 | ± | 1.6 | 4.0 | ± | 1.4 | 4.2 | ± | 1.3 |  |
|  | PK_2-4 | 4.6 | ± | 1.4 |  | 4.6 | ± | 1.5 | 4.6 | ± | 1.3 | 4.6 | ± | 1.2 |  |
|  | NE_2-1 | 4.7 | ± | 1.5 |  | 4.8 | ± | 1.5 | 4.7 | ± | 1.5 | 4.6 | ± | 1.4 |  |
|  | NE_2-2 | 4.7 | ± | 1.6 |  | 4.8 | ± | 1.7 | 4.7 | ± | 1.5 | 4.5 | ± | 1.5 |  |
|  | NE_2-3 | 4.6 | ± | 1.5 |  | 4.7 | ± | 1.6 | 4.7 | ± | 1.5 | 4.5 | ± | 1.4 |  |
|  | NE_2-4 | 4.7 | ± | 1.5 |  | 4.8 | ± | 1.6 | 4.8 | ± | 1.5 | 4.6 | ± | 1.4 |  |
| **Area 3** | PK_3-1 | 4.4 | ± | 1.2 |  | 4.3 | ± | 1.2 | 4.3 | ± | 1.3 | 4.4 | ± | 1.1 |  |
|  | PK_3-2 | 4.7 | ± | 1.2 |  | 4.7 | ± | 1.2 | 4.7 | ± | 1.3 | 4.6 | ± | 1.2 |  |
|  | PK_3-3 | 4.7 | ± | 1.2 |  | 4.7 | ± | 1.2 | 4.7 | ± | 1.2 | 4.6 | ± | 1.1 |  |
|  | PK_3-4 | 4.8 | ± | 1.2 |  | 4.8 | ± | 1.2 | 4.8 | ± | 1.3 | 4.7 | ± | 1.1 |  |
|  | PK_3-5 | 4.9 | ± | 1.3 |  | 5.1 | ± | 1.3 | 4.8 | ± | 1.4 | 4.8 | ± | 1.3 |  |
|  | NE_3-1 | 4.5 | ± | 1.4 |  | 4.7 | ± | 1.5 | 4.4 | ± | 1.5 | 4.3 | ± | 1.4 | * |
|  | NE_3-2 | 4.6 | ± | 1.5 |  | 4.8 | ± | 1.5 | 4.4 | ± | 1.5 | 4.4 | ± | 1.4 | * |
|  | NE_3-3 | 4.6 | ± | 1.5 |  | 4.7 | ± | 1.5 | 4.5 | ± | 1.6 | 4.5 | ± | 1.3 |  |
|  | NE_3-4 | 4.6 | ± | 1.5 |  | 4.8 | ± | 1.5 | 4.5 | ± | 1.6 | 4.5 | ± | 1.4 |  |
|  | NE_3-5 | 4.6 | ± | 1.6 |  | 4.7 | ± | 1.7 | 4.5 | ± | 1.6 | 4.5 | ± | 1.5 |  |
| **Area 4** | PK_4-1 | 4.8 | ± | 1.2 |  | 4.8 | ± | 1.3 | 4.8 | ± | 1.2 | 4.6 | ± | 1.2 |  |
|  | PK_4-2 | 4.9 | ± | 1.2 |  | 5.0 | ± | 1.3 | 4.8 | ± | 1.2 | 4.7 | ± | 1.2 |  |
|  | PK_4-3 | 4.8 | ± | 1.2 |  | 4.9 | ± | 1.3 | 4.8 | ± | 1.2 | 4.7 | ± | 1.2 |  |
|  | NE_4-1 | 4.8 | ± | 1.5 |  | 4.8 | ± | 1.6 | 4.8 | ± | 1.5 | 4.7 | ± | 1.5 |  |
|  | NE_4-2 | 4.8 | ± | 1.5 |  | 4.8 | ± | 1.6 | 4.9 | ± | 1.5 | 4.7 | ± | 1.5 |  |
|  | NE_4-3 | 4.8 | ± | 1.5 |  | 4.9 | ± | 1.6 | 4.9 | ± | 1.5 | 4.7 | ± | 1.4 |  |
| **Area 5** | PK_5-1 | 4.4 | ± | 1.5 |  | 4.4 | ± | 1.5 | 4.4 | ± | 1.5 | 4.4 | ± | 1.4 |  |
|  | PK_5-2 | 4.5 | ± | 1.4 |  | 4.5 | ± | 1.4 | 4.5 | ± | 1.3 | 4.3 | ± | 1.4 |  |
|  | PK_5-3 | 4.5 | ± | 1.3 |  | 4.5 | ± | 1.3 | 4.6 | ± | 1.3 | 4.4 | ± | 1.3 |  |
|  | PK_5-4 | 4.4 | ± | 1.4 |  | 4.4 | ± | 1.4 | 4.4 | ± | 1.4 | 4.3 | ± | 1.3 |  |
|  | PK_5-5 | 4.6 | ± | 1.3 |  | 4.7 | ± | 1.4 | 4.6 | ± | 1.3 | 4.5 | ± | 1.3 |  |
|  | PK_5-6 | 4.6 | ± | 1.3 |  | 4.7 | ± | 1.4 | 4.7 | ± | 1.4 | 4.5 | ± | 1.3 |  |
|  | PK_5-7 | 4.2 | ± | 1.5 |  | 4.3 | ± | 1.5 | 4.2 | ± | 1.5 | 4.0 | ± | 1.4 |  |
|  | PK_5-8 | 4.3 | ± | 1.4 |  | 4.4 | ± | 1.5 | 4.4 | ± | 1.4 | 4.1 | ± | 1.4 |  |
|  | NE_5-1 | 4.8 | ± | 1.5 |  | 4.9 | ± | 1.5 | 4.8 | ± | 1.5 | 4.7 | ± | 1.4 |  |
|  | NE_5-2 | 4.8 | ± | 1.4 |  | 4.9 | ± | 1.5 | 4.8 | ± | 1.5 | 4.7 | ± | 1.3 |  |
|  | NE_5-3 | 4.8 | ± | 1.4 |  | 4.9 | ± | 1.5 | 4.7 | ± | 1.5 | 4.6 | ± | 1.3 |  |
|  | NE_5-4 | 4.7 | ± | 1.5 |  | 4.8 | ± | 1.6 | 4.7 | ± | 1.6 | 4.6 | ± | 1.3 |  |
|  | NE_5-5 | 4.8 | ± | 1.5 |  | 5.0 | ± | 1.5 | 4.8 | ± | 1.5 | 4.6 | ± | 1.4 | * |
|  | NE_5-6 | 4.8 | ± | 1.5 |  | 5.0 | ± | 1.5 | 4.8 | ± | 1.5 | 4.7 | ± | 1.3 |  |
|  | NE_5-7 | 4.9 | ± | 1.6 |  | 5.0 | ± | 1.6 | 4.8 | ± | 1.7 | 4.7 | ± | 1.5 |  |
|  | NE_5-8 | 4.9 | ± | 1.5 |  | 5.0 | ± | 1.6 | 4.9 | ± | 1.6 | 4.8 | ± | 1.4 |  |
| **Area 6** | PK_6-1 | 4.7 | ± | 1.5 |  | 4.7 | ± | 1.6 | 4.5 | ± | 1.5 | 4.9 | ± | 1.3 |  |
|  | PK_6-2 | 5.0 | ± | 1.3 |  | 5.0 | ± | 1.3 | 5.0 | ± | 1.3 | 4.9 | ± | 1.3 |  |
|  | PK_6-3 | 5.1 | ± | 1.3 |  | 5.2 | ± | 1.3 | 5.1 | ± | 1.3 | 5.0 | ± | 1.3 |  |
|  | PK_6-4 | 4.9 | ± | 1.3 |  | 5.0 | ± | 1.3 | 4.8 | ± | 1.3 | 4.9 | ± | 1.2 |  |
|  | PK_6-5 | 4.9 | ± | 1.3 |  | 5.0 | ± | 1.4 | 4.8 | ± | 1.3 | 4.9 | ± | 1.3 |  |
|  | PK_6-6 | 4.9 | ± | 1.4 |  | 5.0 | ± | 1.5 | 4.9 | ± | 1.3 | 4.8 | ± | 1.3 |  |
|  | PK_6-7 | 4.9 | ± | 1.3 |  | 4.9 | ± | 1.4 | 4.9 | ± | 1.2 | 4.8 | ± | 1.3 |  |
|  | PK_6-8 | 4.8 | ± | 1.4 |  | 4.9 | ± | 1.5 | 4.7 | ± | 1.3 | 4.7 | ± | 1.3 |  |
|  | NE_6-1 | 4.8 | ± | 1.6 |  | 5.0 | ± | 1.6 | 4.8 | ± | 1.6 | 4.7 | ± | 1.6 |  |
|  | NE_6-2 | 4.7 | ± | 1.5 |  | 4.8 | ± | 1.6 | 4.7 | ± | 1.5 | 4.6 | ± | 1.5 |  |
|  | NE_6-3 | 4.7 | ± | 1.5 |  | 4.9 | ± | 1.6 | 4.7 | ± | 1.5 | 4.6 | ± | 1.5 |  |
|  | NE_6-4 | 4.7 | ± | 1.5 |  | 4.9 | ± | 1.6 | 4.7 | ± | 1.5 | 4.6 | ± | 1.5 |  |
|  | NE_6-5 | 4.8 | ± | 1.5 |  | 4.9 | ± | 1.6 | 4.9 | ± | 1.5 | 4.7 | ± | 1.5 |  |
|  | NE_6-6 | 4.8 | ± | 1.6 |  | 4.9 | ± | 1.7 | 4.9 | ± | 1.6 | 4.7 | ± | 1.6 |  |
|  | NE_6-7 | 4.8 | ± | 1.6 |  | 4.9 | ± | 1.6 | 4.8 | ± | 1.6 | 4.7 | ± | 1.5 |  |
|  | NE_6-8 | 4.8 | ± | 1.6 |  | 5.0 | ± | 1.6 | 4.9 | ± | 1.5 | 4.7 | ± | 1.5 |  |
|  |  |  |  |  |  | * (<40 yr vs ≥50 yr) | | | | | | | | | |
|  |  |  |  |  |  |  |  |  |  |  |  |  |  |  |  |

Follows

|  |  | **Highest academic attainment** | | | | | | | | | |  | **Judo level** | | | | | | |
| --- | --- | --- | --- | --- | --- | --- | --- | --- | --- | --- | --- | --- | --- | --- | --- | --- | --- | --- | --- |
| **Variables** |  | **≤EQF 5** | | | **EQF 6** | | | **≥EQF 7** | | |  |  | **≤3rd dan** | | | **≥4th dan** | | |  |
| **General** | PK | 4.5 | ± | 1.0 | 4.6 | ± | 1.0 | 4.7 | ± | 0.9 |  |  | 4.5 | ± | 1.0 | 4.7 | ± | 0.9 | * |
|  | NE | 4.6 | ± | 1.2 | 4.8 | ± | 1.2 | 4.8 | ± | 1.2 |  |  | 4.8 | ± | 1.2 | 4.6 | ± | 1.2 | * |
| **General area** | Area1_PK | 4.4 | ± | 1.4 | 4.5 | ± | 1.4 | 4.6 | ± | 1.3 |  |  | 4.5 | ± | 1.5 | 4.6 | ± | 1.3 |  |
|  | Area1_NE | 4.4 | ± | 1.5 | 4.8 | ± | 1.5 | 4.7 | ± | 1.5 |  |  | 4.7 | ± | 1.5 | 4.5 | ± | 1.4 |  |
|  | Area2_PK | 4.2 | ± | 1.2 | 4.3 | ± | 1.3 | 4.6 | ± | 1.2 | # |  | 4.3 | ± | 1.3 | 4.5 | ± | 1.1 | * |
|  | Area2_NE | 4.6 | ± | 1.3 | 4.7 | ± | 1.5 | 4.7 | ± | 1.4 |  |  | 4.7 | ± | 1.5 | 4.6 | ± | 1.4 |  |
|  | Area3_PK | 4.7 | ± | 1.1 | 4.6 | ± | 1.2 | 4.8 | ± | 1.0 |  |  | 4.7 | ± | 1.1 | 4.7 | ± | 1.1 |  |
|  | Area3_NE | 4.4 | ± | 1.4 | 4.7 | ± | 1.4 | 4.6 | ± | 1.4 |  |  | 4.6 | ± | 1.4 | 4.5 | ± | 1.4 |  |
|  | Area4_PK | 4.6 | ± | 1.1 | 4.9 | ± | 1.2 | 5.0 | ± | 1.1 | # |  | 4.8 | ± | 1.2 | 4.9 | ± | 1.1 |  |
|  | Area4_NE | 4.7 | ± | 1.4 | 4.8 | ± | 1.5 | 4.8 | ± | 1.5 |  |  | 4.9 | ± | 1.5 | 4.7 | ± | 1.5 |  |
|  | Area5_PK | 4.3 | ± | 1.2 | 4.5 | ± | 1.3 | 4.5 | ± | 1.1 |  |  | 4.4 | ± | 1.3 | 4.5 | ± | 1.1 | * |
|  | Area5_NE | 4.6 | ± | 1.3 | 4.9 | ± | 1.5 | 4.9 | ± | 1.3 |  |  | 4.9 | ± | 1.4 | 4.7 | ± | 1.3 | * |
|  | Area6_PK | 4.8 | ± | 1.3 | 4.9 | ± | 1.2 | 5.0 | ± | 1.2 |  |  | 4.8 | ± | 1.3 | 5.0 | ± | 1.2 | * |
|  | Area6_NE | 4.7 | ± | 1.5 | 4.8 | ± | 1.6 | 4.9 | ± | 1.5 |  |  | 4.9 | ± | 1.5 | 4.6 | ± | 1.5 | * |
| **Area 1** | PK_1-1 | 4.4 | ± | 1.4 | 4.6 | ± | 1.5 | 4.9 | ± | 1.6 | # |  | 4.6 | ± | 1.6 | 4.8 | ± | 1.5 |  |
|  | PK_1-2 | 4.2 | ± | 1.5 | 4.1 | ± | 1.6 | 4.2 | ± | 1.4 |  |  | 4.2 | ± | 1.6 | 4.2 | ± | 1.4 |  |
|  | PK_1-3 | 4.2 | ± | 1.6 | 4.2 | ± | 1.6 | 4.3 | ± | 1.5 |  |  | 4.2 | ± | 1.7 | 4.2 | ± | 1.5 |  |
|  | PK_1-4 | 4.4 | ± | 1.5 | 4.4 | ± | 1.6 | 4.5 | ± | 1.5 |  |  | 4.4 | ± | 1.6 | 4.5 | ± | 1.5 |  |
|  | PK_1-5 | 4.5 | ± | 1.5 | 4.6 | ± | 1.5 | 4.7 | ± | 1.5 |  |  | 4.5 | ± | 1.6 | 4.6 | ± | 1.4 |  |
|  | PK_1-6 | 4.7 | ± | 1.5 | 4.8 | ± | 1.5 | 5.1 | ± | 1.5 | # |  | 4.8 | ± | 1.5 | 5.0 | ± | 1.4 |  |
|  | PK_1-7 | 4.5 | ± | 1.5 | 4.6 | ± | 1.5 | 4.7 | ± | 1.5 |  |  | 4.6 | ± | 1.6 | 4.7 | ± | 1.4 |  |
|  | NE_1-1 | 4.5 | ± | 1.5 | 4.8 | ± | 1.6 | 4.6 | ± | 1.7 |  |  | 4.7 | ± | 1.6 | 4.5 | ± | 1.6 |  |
|  | NE_1-2 | 4.3 | ± | 1.6 | 4.7 | ± | 1.6 | 4.7 | ± | 1.5 | # |  | 4.7 | ± | 1.6 | 4.4 | ± | 1.5 |  |
|  | NE_1-3 | 4.3 | ± | 1.6 | 4.7 | ± | 1.6 | 4.7 | ± | 1.5 |  |  | 4.7 | ± | 1.6 | 4.4 | ± | 1.6 |  |
|  | NE_1-4 | 4.4 | ± | 1.6 | 4.8 | ± | 1.5 | 4.8 | ± | 1.5 |  |  | 4.7 | ± | 1.6 | 4.5 | ± | 1.5 |  |
|  | NE_1-5 | 4.5 | ± | 1.6 | 4.9 | ± | 1.6 | 4.7 | ± | 1.6 |  |  | 4.8 | ± | 1.6 | 4.6 | ± | 1.5 |  |
|  | NE_1-6 | 4.6 | ± | 1.6 | 4.9 | ± | 1.6 | 4.6 | ± | 1.7 |  |  | 4.9 | ± | 1.6 | 4.5 | ± | 1.6 | * |
|  | NE_1-7 | 4.4 | ± | 1.5 | 4.8 | ± | 1.6 | 4.6 | ± | 1.6 |  |  | 4.7 | ± | 1.6 | 4.5 | ± | 1.5 |  |
| **Area 2** | PK_2-1 | 4.6 | ± | 1.2 | 4.7 | ± | 1.3 | 5.0 | ± | 1.2 | # |  | 4.7 | ± | 1.4 | 4.9 | ± | 1.1 |  |
|  | PK_2-2 | 3.9 | ± | 1.4 | 4.0 | ± | 1.6 | 4.2 | ± | 1.5 |  |  | 3.9 | ± | 1.6 | 4.2 | ± | 1.4 | * |
|  | PK_2-3 | 3.9 | ± | 1.5 | 4.1 | ± | 1.6 | 4.3 | ± | 1.4 | # |  | 4.0 | ± | 1.6 | 4.3 | ± | 1.4 |  |
|  | PK_2-4 | 4.4 | ± | 1.3 | 4.6 | ± | 1.5 | 4.8 | ± | 1.2 | # |  | 4.5 | ± | 1.5 | 4.8 | ± | 1.2 | * |
|  | NE_2-1 | 4.7 | ± | 1.4 | 4.8 | ± | 1.6 | 4.7 | ± | 1.6 |  |  | 4.8 | ± | 1.5 | 4.6 | ± | 1.5 |  |
|  | NE_2-2 | 4.5 | ± | 1.4 | 4.7 | ± | 1.7 | 4.8 | ± | 1.6 |  |  | 4.7 | ± | 1.6 | 4.6 | ± | 1.5 |  |
|  | NE_2-3 | 4.5 | ± | 1.5 | 4.7 | ± | 1.5 | 4.7 | ± | 1.5 |  |  | 4.7 | ± | 1.6 | 4.5 | ± | 1.4 |  |
|  | NE_2-4 | 4.6 | ± | 1.4 | 4.8 | ± | 1.6 | 4.8 | ± | 1.6 |  |  | 4.8 | ± | 1.6 | 4.7 | ± | 1.5 |  |
| **Area 3** | PK_3-1 | 4.3 | ± | 1.2 | 4.3 | ± | 1.3 | 4.4 | ± | 1.2 |  |  | 4.3 | ± | 1.3 | 4.4 | ± | 1.2 |  |
|  | PK_3-2 | 4.6 | ± | 1.3 | 4.6 | ± | 1.3 | 4.8 | ± | 1.0 |  |  | 4.7 | ± | 1.2 | 4.7 | ± | 1.2 |  |
|  | PK_3-3 | 4.7 | ± | 1.2 | 4.6 | ± | 1.3 | 4.8 | ± | 1.1 |  |  | 4.7 | ± | 1.2 | 4.7 | ± | 1.1 |  |
|  | PK_3-4 | 4.7 | ± | 1.2 | 4.8 | ± | 1.3 | 4.9 | ± | 1.1 |  |  | 4.8 | ± | 1.2 | 4.8 | ± | 1.2 |  |
|  | PK_3-5 | 4.9 | ± | 1.3 | 4.9 | ± | 1.5 | 4.9 | ± | 1.2 |  |  | 4.9 | ± | 1.4 | 4.9 | ± | 1.3 |  |
|  | NE_3-1 | 4.3 | ± | 1.4 | 4.6 | ± | 1.4 | 4.5 | ± | 1.5 |  |  | 4.5 | ± | 1.5 | 4.4 | ± | 1.4 |  |
|  | NE_3-2 | 4.4 | ± | 1.5 | 4.7 | ± | 1.5 | 4.6 | ± | 1.4 |  |  | 4.6 | ± | 1.5 | 4.5 | ± | 1.5 |  |
|  | NE_3-3 | 4.5 | ± | 1.5 | 4.7 | ± | 1.4 | 4.6 | ± | 1.5 |  |  | 4.7 | ± | 1.5 | 4.5 | ± | 1.4 |  |
|  | NE_3-4 | 4.4 | ± | 1.5 | 4.7 | ± | 1.5 | 4.6 | ± | 1.5 |  |  | 4.7 | ± | 1.5 | 4.5 | ± | 1.5 |  |
|  | NE_3-5 | 4.5 | ± | 1.6 | 4.7 | ± | 1.6 | 4.5 | ± | 1.6 |  |  | 4.7 | ± | 1.6 | 4.4 | ± | 1.6 |  |
| **Area 4** | PK_4-1 | 4.6 | ± | 1.2 | 4.8 | ± | 1.3 | 4.9 | ± | 1.2 | # |  | 4.7 | ± | 1.3 | 4.8 | ± | 1.2 |  |
|  | PK_4-2 | 4.6 | ± | 1.2 | 4.9 | ± | 1.3 | 5.0 | ± | 1.2 | * # |  | 4.8 | ± | 1.3 | 4.9 | ± | 1.2 |  |
|  | PK_4-3 | 4.6 | ± | 1.1 | 4.9 | ± | 1.3 | 4.9 | ± | 1.2 |  |  | 4.8 | ± | 1.2 | 4.9 | ± | 1.2 |  |
|  | NE_4-1 | 4.6 | ± | 1.5 | 4.8 | ± | 1.5 | 4.8 | ± | 1.5 |  |  | 4.8 | ± | 1.5 | 4.7 | ± | 1.5 |  |
|  | NE_4-2 | 4.8 | ± | 1.5 | 4.8 | ± | 1.5 | 4.9 | ± | 1.5 |  |  | 4.9 | ± | 1.5 | 4.8 | ± | 1.5 |  |
|  | NE_4-3 | 4.7 | ± | 1.5 | 4.9 | ± | 1.5 | 4.9 | ± | 1.5 |  |  | 4.9 | ± | 1.5 | 4.7 | ± | 1.5 |  |
| **Area 5** | PK_5-1 | 4.4 | ± | 1.4 | 4.4 | ± | 1.5 | 4.5 | ± | 1.5 |  |  | 4.4 | ± | 1.5 | 4.5 | ± | 1.4 |  |
|  | PK_5-2 | 4.3 | ± | 1.4 | 4.5 | ± | 1.4 | 4.6 | ± | 1.3 |  |  | 4.4 | ± | 1.4 | 4.6 | ± | 1.3 |  |
|  | PK_5-3 | 4.5 | ± | 1.4 | 4.5 | ± | 1.3 | 4.6 | ± | 1.3 |  |  | 4.4 | ± | 1.4 | 4.6 | ± | 1.2 |  |
|  | PK_5-4 | 4.3 | ± | 1.4 | 4.4 | ± | 1.5 | 4.4 | ± | 1.3 |  |  | 4.2 | ± | 1.5 | 4.5 | ± | 1.3 | * |
|  | PK_5-5 | 4.6 | ± | 1.4 | 4.7 | ± | 1.4 | 4.6 | ± | 1.3 |  |  | 4.6 | ± | 1.4 | 4.7 | ± | 1.2 |  |
|  | PK_5-6 | 4.5 | ± | 1.4 | 4.7 | ± | 1.4 | 4.7 | ± | 1.2 |  |  | 4.6 | ± | 1.4 | 4.7 | ± | 1.2 |  |
|  | PK_5-7 | 4.0 | ± | 1.5 | 4.3 | ± | 1.5 | 4.2 | ± | 1.3 |  |  | 4.1 | ± | 1.6 | 4.3 | ± | 1.3 |  |
|  | PK_5-8 | 4.1 | ± | 1.5 | 4.4 | ± | 1.5 | 4.3 | ± | 1.3 |  |  | 4.2 | ± | 1.5 | 4.4 | ± | 1.4 |  |
|  | NE_5-1 | 4.7 | ± | 1.4 | 4.9 | ± | 1.6 | 4.8 | ± | 1.4 |  |  | 4.9 | ± | 1.5 | 4.7 | ± | 1.4 |  |
|  | NE_5-2 | 4.6 | ± | 1.4 | 4.9 | ± | 1.5 | 4.9 | ± | 1.3 |  |  | 4.9 | ± | 1.5 | 4.7 | ± | 1.4 | * |
|  | NE_5-3 | 4.6 | ± | 1.4 | 4.8 | ± | 1.5 | 4.8 | ± | 1.3 |  |  | 4.9 | ± | 1.5 | 4.6 | ± | 1.4 |  |
|  | NE_5-4 | 4.5 | ± | 1.5 | 4.8 | ± | 1.7 | 4.7 | ± | 1.4 |  |  | 4.8 | ± | 1.6 | 4.6 | ± | 1.4 |  |
|  | NE_5-5 | 4.6 | ± | 1.4 | 4.8 | ± | 1.6 | 4.9 | ± | 1.4 |  |  | 4.9 | ± | 1.5 | 4.7 | ± | 1.4 | * |
|  | NE_5-6 | 4.7 | ± | 1.4 | 5.0 | ± | 1.6 | 4.9 | ± | 1.4 |  |  | 5.0 | ± | 1.5 | 4.7 | ± | 1.4 | * |
|  | NE_5-7 | 4.6 | ± | 1.6 | 5.0 | ± | 1.6 | 4.9 | ± | 1.5 |  |  | 5.0 | ± | 1.7 | 4.7 | ± | 1.5 | * |
|  | NE_5-8 | 4.7 | ± | 1.6 | 5.0 | ± | 1.6 | 5.0 | ± | 1.4 |  |  | 5.0 | ± | 1.6 | 4.8 | ± | 1.5 | * |
| **Area 6** | PK_6-1 | 4.7 | ± | 1.4 | 4.7 | ± | 1.6 | 4.7 | ± | 1.5 |  |  | 4.6 | ± | 1.6 | 4.9 | ± | 1.4 | * |
|  | PK_6-2 | 4.9 | ± | 1.3 | 5.0 | ± | 1.3 | 5.0 | ± | 1.3 |  |  | 4.9 | ± | 1.3 | 5.0 | ± | 1.3 |  |
|  | PK_6-3 | 5.0 | ± | 1.4 | 5.1 | ± | 1.2 | 5.1 | ± | 1.3 |  |  | 5.0 | ± | 1.3 | 5.2 | ± | 1.3 |  |
|  | PK_6-4 | 4.8 | ± | 1.4 | 4.9 | ± | 1.3 | 5.0 | ± | 1.3 |  |  | 4.8 | ± | 1.3 | 5.0 | ± | 1.3 | * |
|  | PK_6-5 | 4.8 | ± | 1.4 | 4.9 | ± | 1.3 | 5.0 | ± | 1.3 |  |  | 4.8 | ± | 1.4 | 5.0 | ± | 1.2 |  |
|  | PK_6-6 | 4.7 | ± | 1.5 | 5.0 | ± | 1.4 | 5.0 | ± | 1.3 |  |  | 4.8 | ± | 1.4 | 5.0 | ± | 1.3 | * |
|  | PK_6-7 | 4.8 | ± | 1.4 | 4.9 | ± | 1.3 | 4.9 | ± | 1.2 |  |  | 4.8 | ± | 1.4 | 5.0 | ± | 1.2 |  |
|  | PK_6-8 | 4.7 | ± | 1.5 | 4.8 | ± | 1.3 | 4.9 | ± | 1.4 |  |  | 4.7 | ± | 1.4 | 4.9 | ± | 1.3 |  |
|  | NE_6-1 | 4.6 | ± | 1.5 | 4.9 | ± | 1.6 | 5.0 | ± | 1.6 |  |  | 5.0 | ± | 1.6 | 4.7 | ± | 1.6 | * |
|  | NE_6-2 | 4.6 | ± | 1.5 | 4.7 | ± | 1.6 | 4.9 | ± | 1.5 |  |  | 4.8 | ± | 1.5 | 4.6 | ± | 1.5 |  |
|  | NE_6-3 | 4.6 | ± | 1.5 | 4.7 | ± | 1.6 | 4.8 | ± | 1.5 |  |  | 4.9 | ± | 1.5 | 4.5 | ± | 1.6 | * |
|  | NE_6-4 | 4.6 | ± | 1.5 | 4.7 | ± | 1.6 | 4.8 | ± | 1.5 |  |  | 4.9 | ± | 1.5 | 4.5 | ± | 1.5 | * |
|  | NE_6-5 | 4.7 | ± | 1.5 | 4.8 | ± | 1.6 | 4.9 | ± | 1.5 |  |  | 5.0 | ± | 1.5 | 4.6 | ± | 1.5 | * |
|  | NE_6-6 | 4.7 | ± | 1.6 | 4.8 | ± | 1.6 | 5.0 | ± | 1.6 |  |  | 5.0 | ± | 1.6 | 4.6 | ± | 1.6 | * |
|  | NE_6-7 | 4.7 | ± | 1.6 | 4.8 | ± | 1.6 | 4.9 | ± | 1.5 |  |  | 4.9 | ± | 1.5 | 4.6 | ± | 1.6 | * |
|  | NE_6-8 | 4.7 | ± | 1.5 | 4.9 | ± | 1.6 | 4.9 | ± | 1.5 |  |  | 5.0 | ± | 1.5 | 4.6 | ± | 1.5 | * |
|  |  | * (≤EQF 5 vs EQF 6); # (≤EQF 5 vs ≥EQF 7) | | | | | | | | | |  |  |  |  |  |  |  |  |
|  |  |  |  |  |  |  |  |  |  |  |  |  |  |  |  |  |  |  |  |

Follows

|  |  | **Former competition level** | | | | | | |  | **Judo education level** | | | | | | | | | |
| --- | --- | --- | --- | --- | --- | --- | --- | --- | --- | --- | --- | --- | --- | --- | --- | --- | --- | --- | --- |
| **Variables** |  | **Elite** | | | **Non-elite** | | |  |  | **Int-1** | | | **Int-2** | | | **Diff-Qual** | | |  |
| **General** | PK | 4.7 | ± | 0.9 | 4.5 | ± | 1.0 | * |  | 4.6 | ± | 1.0 | 4.7 | ± | 1.0 | 4.6 | ± | 1.0 |  |
|  | NE | 4.7 | ± | 1.2 | 4.7 | ± | 1.3 |  |  | 4.8 | ± | 1.2 | 4.6 | ± | 1.3 | 4.6 | ± | 1.1 |  |
| **General area** | Area1_PK | 4.6 | ± | 1.4 | 4.4 | ± | 1.4 |  |  | 4.4 | ± | 1.4 | 4.7 | ± | 1.3 | 4.4 | ± | 1.4 |  |
|  | Area1_NE | 4.7 | ± | 1.5 | 4.6 | ± | 1.4 |  |  | 4.7 | ± | 1.5 | 4.5 | ± | 1.5 | 4.5 | ± | 1.4 |  |
|  | Area2_PK | 4.5 | ± | 1.2 | 4.2 | ± | 1.3 | * |  | 4.4 | ± | 1.3 | 4.4 | ± | 1.2 | 4.3 | ± | 1.3 |  |
|  | Area2_NE | 4.7 | ± | 1.4 | 4.6 | ± | 1.4 |  |  | 4.7 | ± | 1.4 | 4.6 | ± | 1.4 | 4.6 | ± | 1.3 |  |
|  | Area3_PK | 4.7 | ± | 1.1 | 4.6 | ± | 1.1 |  |  | 4.6 | ± | 1.1 | 4.8 | ± | 1.1 | 4.7 | ± | 1.0 |  |
|  | Area3_NE | 4.6 | ± | 1.3 | 4.4 | ± | 1.5 |  |  | 4.6 | ± | 1.4 | 4.4 | ± | 1.5 | 4.6 | ± | 1.3 |  |
|  | Area4_PK | 4.9 | ± | 1.1 | 4.7 | ± | 1.2 | * |  | 4.8 | ± | 1.1 | 4.9 | ± | 1.2 | 4.8 | ± | 1.2 |  |
|  | Area4_NE | 4.8 | ± | 1.4 | 4.8 | ± | 1.5 |  |  | 4.9 | ± | 1.5 | 4.7 | ± | 1.5 | 4.7 | ± | 1.4 |  |
|  | Area5_PK | 4.5 | ± | 1.2 | 4.3 | ± | 1.2 |  |  | 4.4 | ± | 1.2 | 4.4 | ± | 1.2 | 4.5 | ± | 1.2 |  |
|  | Area5_NE | 4.8 | ± | 1.3 | 4.8 | ± | 1.4 |  |  | 4.9 | ± | 1.4 | 4.7 | ± | 1.5 | 4.7 | ± | 1.2 |  |
|  | Area6_PK | 5.0 | ± | 1.2 | 4.8 | ± | 1.3 |  |  | 4.8 | ± | 1.3 | 5.1 | ± | 1.2 | 4.7 | ± | 1.3 |  |
|  | Area6_NE | 4.8 | ± | 1.5 | 4.8 | ± | 1.5 |  |  | 4.9 | ± | 1.4 | 4.7 | ± | 1.6 | 4.7 | ± | 1.3 |  |
| **Area 1** | PK_1-1 | 4.7 | ± | 1.5 | 4.5 | ± | 1.5 |  |  | 4.6 | ± | 1.5 | 4.8 | ± | 1.5 | 4.6 | ± | 1.5 |  |
|  | PK_1-2 | 4.3 | ± | 1.5 | 4.1 | ± | 1.5 |  |  | 4.2 | ± | 1.5 | 4.3 | ± | 1.5 | 4.0 | ± | 1.6 |  |
|  | PK_1-3 | 4.3 | ± | 1.5 | 4.1 | ± | 1.6 |  |  | 4.1 | ± | 1.6 | 4.4 | ± | 1.6 | 4.1 | ± | 1.5 |  |
|  | PK_1-4 | 4.5 | ± | 1.6 | 4.3 | ± | 1.6 |  |  | 4.4 | ± | 1.6 | 4.7 | ± | 1.5 | 4.3 | ± | 1.5 |  |
|  | PK_1-5 | 4.7 | ± | 1.5 | 4.4 | ± | 1.6 | * |  | 4.5 | ± | 1.5 | 4.8 | ± | 1.5 | 4.4 | ± | 1.5 |  |
|  | PK_1-6 | 5.0 | ± | 1.5 | 4.7 | ± | 1.5 | * |  | 4.8 | ± | 1.5 | 5.0 | ± | 1.4 | 4.8 | ± | 1.5 |  |
|  | PK_1-7 | 4.7 | ± | 1.5 | 4.5 | ± | 1.6 |  |  | 4.6 | ± | 1.6 | 4.7 | ± | 1.5 | 4.5 | ± | 1.4 |  |
|  | NE_1-1 | 4.7 | ± | 1.6 | 4.6 | ± | 1.6 |  |  | 4.7 | ± | 1.6 | 4.5 | ± | 1.6 | 4.6 | ± | 1.5 |  |
|  | NE_1-2 | 4.6 | ± | 1.6 | 4.4 | ± | 1.5 |  |  | 4.7 | ± | 1.6 | 4.5 | ± | 1.5 | 4.3 | ± | 1.7 |  |
|  | NE_1-3 | 4.6 | ± | 1.6 | 4.5 | ± | 1.5 |  |  | 4.7 | ± | 1.6 | 4.5 | ± | 1.6 | 4.4 | ± | 1.5 |  |
|  | NE_1-4 | 4.7 | ± | 1.6 | 4.6 | ± | 1.5 |  |  | 4.7 | ± | 1.6 | 4.6 | ± | 1.6 | 4.6 | ± | 1.6 |  |
|  | NE_1-5 | 4.7 | ± | 1.6 | 4.7 | ± | 1.6 |  |  | 4.8 | ± | 1.6 | 4.5 | ± | 1.6 | 4.6 | ± | 1.6 |  |
|  | NE_1-6 | 4.7 | ± | 1.6 | 4.7 | ± | 1.6 |  |  | 4.9 | ± | 1.6 | 4.5 | ± | 1.7 | 4.6 | ± | 1.6 |  |
|  | NE_1-7 | 4.7 | ± | 1.6 | 4.5 | ± | 1.6 |  |  | 4.7 | ± | 1.6 | 4.5 | ± | 1.6 | 4.5 | ± | 1.5 |  |
| **Area 2** | PK_2-1 | 4.8 | ± | 1.2 | 4.7 | ± | 1.3 |  |  | 4.7 | ± | 1.3 | 4.8 | ± | 1.2 | 4.8 | ± | 1.3 |  |
|  | PK_2-2 | 4.1 | ± | 1.4 | 3.9 | ± | 1.5 |  |  | 4.0 | ± | 1.5 | 4.0 | ± | 1.4 | 4.0 | ± | 1.5 |  |
|  | PK_2-3 | 4.2 | ± | 1.4 | 3.9 | ± | 1.6 | * |  | 4.1 | ± | 1.5 | 4.1 | ± | 1.4 | 4.0 | ± | 1.6 |  |
|  | PK_2-4 | 4.7 | ± | 1.3 | 4.4 | ± | 1.4 | * |  | 4.6 | ± | 1.4 | 4.7 | ± | 1.3 | 4.5 | ± | 1.5 |  |
|  | NE_2-1 | 4.8 | ± | 1.5 | 4.7 | ± | 1.5 |  |  | 4.8 | ± | 1.5 | 4.6 | ± | 1.5 | 4.6 | ± | 1.5 |  |
|  | NE_2-2 | 4.7 | ± | 1.6 | 4.6 | ± | 1.6 |  |  | 4.7 | ± | 1.6 | 4.7 | ± | 1.5 | 4.7 | ± | 1.4 |  |
|  | NE_2-3 | 4.7 | ± | 1.5 | 4.6 | ± | 1.5 |  |  | 4.7 | ± | 1.5 | 4.6 | ± | 1.5 | 4.5 | ± | 1.4 |  |
|  | NE_2-4 | 4.8 | ± | 1.5 | 4.6 | ± | 1.6 |  |  | 4.7 | ± | 1.6 | 4.7 | ± | 1.5 | 4.7 | ± | 1.5 |  |
| **Area 3** | PK_3-1 | 4.4 | ± | 1.2 | 4.3 | ± | 1.3 |  |  | 4.3 | ± | 1.3 | 4.5 | ± | 1.2 | 4.3 | ± | 1.1 |  |
|  | PK_3-2 | 4.7 | ± | 1.2 | 4.6 | ± | 1.2 |  |  | 4.7 | ± | 1.2 | 4.7 | ± | 1.2 | 4.6 | ± | 1.2 |  |
|  | PK_3-3 | 4.7 | ± | 1.2 | 4.7 | ± | 1.2 |  |  | 4.7 | ± | 1.2 | 4.8 | ± | 1.2 | 4.6 | ± | 1.2 |  |
|  | PK_3-4 | 4.8 | ± | 1.2 | 4.7 | ± | 1.2 |  |  | 4.8 | ± | 1.2 | 4.9 | ± | 1.2 | 4.7 | ± | 1.2 |  |
|  | PK_3-5 | 4.9 | ± | 1.3 | 4.9 | ± | 1.4 |  |  | 4.8 | ± | 1.4 | 5.0 | ± | 1.3 | 5.1 | ± | 1.2 |  |
|  | NE_3-1 | 4.6 | ± | 1.4 | 4.3 | ± | 1.6 |  |  | 4.5 | ± | 1.5 | 4.4 | ± | 1.4 | 4.5 | ± | 1.3 |  |
|  | NE_3-2 | 4.7 | ± | 1.4 | 4.4 | ± | 1.5 |  |  | 4.6 | ± | 1.5 | 4.5 | ± | 1.5 | 4.6 | ± | 1.4 |  |
|  | NE_3-3 | 4.6 | ± | 1.4 | 4.5 | ± | 1.6 |  |  | 4.6 | ± | 1.5 | 4.5 | ± | 1.5 | 4.6 | ± | 1.3 |  |
|  | NE_3-4 | 4.7 | ± | 1.4 | 4.5 | ± | 1.6 |  |  | 4.6 | ± | 1.5 | 4.5 | ± | 1.5 | 4.6 | ± | 1.4 |  |
|  | NE_3-5 | 4.6 | ± | 1.6 | 4.5 | ± | 1.7 |  |  | 4.7 | ± | 1.6 | 4.4 | ± | 1.7 | 4.5 | ± | 1.6 |  |
| **Area 4** | PK_4-1 | 4.9 | ± | 1.2 | 4.6 | ± | 1.3 | * |  | 4.7 | ± | 1.2 | 4.9 | ± | 1.3 | 4.7 | ± | 1.2 |  |
|  | PK_4-2 | 5.0 | ± | 1.2 | 4.7 | ± | 1.3 | * |  | 4.8 | ± | 1.2 | 4.9 | ± | 1.3 | 4.9 | ± | 1.3 |  |
|  | PK_4-3 | 4.9 | ± | 1.2 | 4.7 | ± | 1.2 |  |  | 4.8 | ± | 1.2 | 4.8 | ± | 1.3 | 4.8 | ± | 1.3 |  |
|  | NE_4-1 | 4.8 | ± | 1.5 | 4.7 | ± | 1.6 |  |  | 4.8 | ± | 1.5 | 4.7 | ± | 1.5 | 4.6 | ± | 1.5 |  |
|  | NE_4-2 | 4.8 | ± | 1.5 | 4.8 | ± | 1.6 |  |  | 4.9 | ± | 1.5 | 4.7 | ± | 1.6 | 4.7 | ± | 1.4 |  |
|  | NE_4-3 | 4.8 | ± | 1.5 | 4.8 | ± | 1.5 |  |  | 4.9 | ± | 1.5 | 4.7 | ± | 1.5 | 4.7 | ± | 1.5 |  |
| **Area 5** | PK_5-1 | 4.5 | ± | 1.5 | 4.3 | ± | 1.4 |  |  | 4.5 | ± | 1.5 | 4.3 | ± | 1.5 | 4.4 | ± | 1.3 |  |
|  | PK_5-2 | 4.6 | ± | 1.3 | 4.3 | ± | 1.4 | * |  | 4.5 | ± | 1.3 | 4.5 | ± | 1.4 | 4.5 | ± | 1.4 |  |
|  | PK_5-3 | 4.6 | ± | 1.3 | 4.4 | ± | 1.4 |  |  | 4.5 | ± | 1.3 | 4.5 | ± | 1.3 | 4.6 | ± | 1.4 |  |
|  | PK_5-4 | 4.4 | ± | 1.4 | 4.2 | ± | 1.4 |  |  | 4.3 | ± | 1.4 | 4.4 | ± | 1.4 | 4.5 | ± | 1.4 |  |
|  | PK_5-5 | 4.7 | ± | 1.3 | 4.5 | ± | 1.4 |  |  | 4.6 | ± | 1.4 | 4.6 | ± | 1.3 | 4.7 | ± | 1.4 |  |
|  | PK_5-6 | 4.7 | ± | 1.3 | 4.5 | ± | 1.4 |  |  | 4.6 | ± | 1.4 | 4.6 | ± | 1.3 | 4.7 | ± | 1.3 |  |
|  | PK_5-7 | 4.3 | ± | 1.5 | 4.0 | ± | 1.5 |  |  | 4.2 | ± | 1.5 | 4.1 | ± | 1.4 | 4.3 | ± | 1.4 |  |
|  | PK_5-8 | 4.4 | ± | 1.5 | 4.2 | ± | 1.4 |  |  | 4.3 | ± | 1.5 | 4.3 | ± | 1.4 | 4.4 | ± | 1.3 |  |
|  | NE_5-1 | 4.8 | ± | 1.5 | 4.8 | ± | 1.5 |  |  | 4.9 | ± | 1.5 | 4.7 | ± | 1.5 | 4.7 | ± | 1.4 |  |
|  | NE_5-2 | 4.8 | ± | 1.4 | 4.8 | ± | 1.4 |  |  | 4.9 | ± | 1.4 | 4.7 | ± | 1.5 | 4.7 | ± | 1.3 |  |
|  | NE_5-3 | 4.8 | ± | 1.4 | 4.7 | ± | 1.4 |  |  | 4.8 | ± | 1.4 | 4.7 | ± | 1.5 | 4.7 | ± | 1.2 |  |
|  | NE_5-4 | 4.7 | ± | 1.5 | 4.7 | ± | 1.6 |  |  | 4.8 | ± | 1.5 | 4.6 | ± | 1.6 | 4.7 | ± | 1.4 |  |
|  | NE_5-5 | 4.9 | ± | 1.4 | 4.7 | ± | 1.5 |  |  | 4.9 | ± | 1.5 | 4.7 | ± | 1.5 | 4.7 | ± | 1.3 |  |
|  | NE_5-6 | 4.9 | ± | 1.4 | 4.8 | ± | 1.5 |  |  | 4.9 | ± | 1.4 | 4.7 | ± | 1.5 | 4.7 | ± | 1.3 |  |
|  | NE_5-7 | 4.8 | ± | 1.5 | 4.9 | ± | 1.7 |  |  | 4.9 | ± | 1.6 | 4.7 | ± | 1.7 | 4.9 | ± | 1.4 |  |
|  | NE_5-8 | 4.9 | ± | 1.5 | 4.9 | ± | 1.6 |  |  | 4.9 | ± | 1.5 | 4.9 | ± | 1.6 | 4.8 | ± | 1.4 |  |
| **Area 6** | PK_6-1 | 4.7 | ± | 1.5 | 4.6 | ± | 1.5 |  |  | 4.7 | ± | 1.5 | 4.8 | ± | 1.5 | 4.5 | ± | 1.4 |  |
|  | PK_6-2 | 5.0 | ± | 1.3 | 4.9 | ± | 1.3 |  |  | 4.9 | ± | 1.4 | 5.1 | ± | 1.2 | 4.7 | ± | 1.3 |  |
|  | PK_6-3 | 5.1 | ± | 1.3 | 5.0 | ± | 1.3 |  |  | 5.0 | ± | 1.3 | 5.2 | ± | 1.2 | 5.1 | ± | 1.2 |  |
|  | PK_6-4 | 5.0 | ± | 1.3 | 4.8 | ± | 1.3 | * |  | 4.9 | ± | 1.3 | 5.1 | ± | 1.2 | 4.7 | ± | 1.4 |  |
|  | PK_6-5 | 5.0 | ± | 1.3 | 4.7 | ± | 1.4 | * |  | 4.8 | ± | 1.3 | 5.1 | ± | 1.2 | 4.7 | ± | 1.5 |  |
|  | PK_6-6 | 5.0 | ± | 1.4 | 4.8 | ± | 1.4 |  |  | 4.8 | ± | 1.4 | 5.1 | ± | 1.3 | 4.7 | ± | 1.5 |  |
|  | PK_6-7 | 5.0 | ± | 1.3 | 4.8 | ± | 1.3 |  |  | 4.8 | ± | 1.3 | 5.1 | ± | 1.3 | 4.7 | ± | 1.4 | * |
|  | PK_6-8 | 5.0 | ± | 1.4 | 4.6 | ± | 1.4 | * |  | 4.8 | ± | 1.4 | 5.0 | ± | 1.3 | 4.6 | ± | 1.5 |  |
|  | NE_6-1 | 4.8 | ± | 1.6 | 4.9 | ± | 1.6 |  |  | 4.9 | ± | 1.6 | 4.8 | ± | 1.7 | 4.8 | ± | 1.5 |  |
|  | NE_6-2 | 4.7 | ± | 1.5 | 4.7 | ± | 1.6 |  |  | 4.8 | ± | 1.5 | 4.6 | ± | 1.6 | 4.7 | ± | 1.5 |  |
|  | NE_6-3 | 4.7 | ± | 1.5 | 4.7 | ± | 1.6 |  |  | 4.8 | ± | 1.5 | 4.6 | ± | 1.7 | 4.6 | ± | 1.4 |  |
|  | NE_6-4 | 4.7 | ± | 1.5 | 4.8 | ± | 1.5 |  |  | 4.8 | ± | 1.5 | 4.6 | ± | 1.7 | 4.6 | ± | 1.4 |  |
|  | NE_6-5 | 4.8 | ± | 1.5 | 4.9 | ± | 1.6 |  |  | 4.9 | ± | 1.5 | 4.8 | ± | 1.7 | 4.7 | ± | 1.4 |  |
|  | NE_6-6 | 4.8 | ± | 1.6 | 4.9 | ± | 1.7 |  |  | 4.9 | ± | 1.6 | 4.8 | ± | 1.8 | 4.9 | ± | 1.4 |  |
|  | NE_6-7 | 4.8 | ± | 1.5 | 4.8 | ± | 1.6 |  |  | 4.9 | ± | 1.5 | 4.7 | ± | 1.7 | 4.7 | ± | 1.4 |  |
|  | NE_6-8 | 4.8 | ± | 1.5 | 4.9 | ± | 1.6 |  |  | 4.9 | ± | 1.5 | 4.7 | ± | 1.7 | 4.9 | ± | 1.4 |  |
|  |  |  |  |  |  |  |  |  |  | * (Int-1 vs Diff-Qual) | | | | | | | | | |
|  |  |  |  |  |  |  |  |  |  |  |  |  |  |  |  |  |  |  |  |

Follows

|  |  | **Coaching experience** | | | | | | | | | |  | **Coaching older judo pract.** | | | | | | |  | **Salary for judo coaching** | | | | | | |
| --- | --- | --- | --- | --- | --- | --- | --- | --- | --- | --- | --- | --- | --- | --- | --- | --- | --- | --- | --- | --- | --- | --- | --- | --- | --- | --- | --- |
| **Variables** |  | **<10 years** | | | **10-20 years** | | | **≥21 years** | | |  |  | **Yes** | | | **No** | | |  |  | **Paid** | | | **Volunteer** | | |  |
| **General** | PK | 4.6 | ± | 0.9 | 4.4 | ± | 1.0 | 4.8 | ± | 0.9 | ¥ |  | 4.8 | ± | 1.0 | 4.5 | ± | 1.0 | * |  | 4.6 | ± | 1.0 | 4.6 | ± | 0.9 |  |
|  | NE | 4.9 | ± | 1.2 | 4.6 | ± | 1.3 | 4.6 | ± | 1.2 | * # |  | 4.6 | ± | 1.3 | 4.8 | ± | 1.2 | * |  | 4.8 | ± | 1.2 | 4.6 | ± | 1.2 |  |
| **General area** | Area1_PK | 4.4 | ± | 1.4 | 4.3 | ± | 1.4 | 4.8 | ± | 1.3 | # ¥ |  | 4.6 | ± | 1.4 | 4.5 | ± | 1.4 |  |  | 4.4 | ± | 1.4 | 4.6 | ± | 1.3 |  |
|  | Area1_NE | 4.9 | ± | 1.4 | 4.5 | ± | 1.5 | 4.5 | ± | 1.4 | # |  | 4.5 | ± | 1.5 | 4.7 | ± | 1.4 |  |  | 4.8 | ± | 1.5 | 4.5 | ± | 1.4 | * |
|  | Area2_PK | 4.4 | ± | 1.3 | 4.3 | ± | 1.2 | 4.5 | ± | 1.3 |  |  | 4.4 | ± | 1.3 | 4.3 | ± | 1.2 |  |  | 4.4 | ± | 1.3 | 4.4 | ± | 1.2 |  |
|  | Area2_NE | 5.0 | ± | 1.4 | 4.6 | ± | 1.4 | 4.6 | ± | 1.4 | * # |  | 4.5 | ± | 1.5 | 4.8 | ± | 1.4 |  |  | 4.9 | ± | 1.4 | 4.5 | ± | 1.4 | * |
|  | Area3_PK | 4.7 | ± | 1.0 | 4.5 | ± | 1.2 | 4.8 | ± | 1.1 |  |  | 4.8 | ± | 1.1 | 4.6 | ± | 1.1 | * |  | 4.7 | ± | 1.1 | 4.7 | ± | 1.0 |  |
|  | Area3_NE | 4.7 | ± | 1.4 | 4.4 | ± | 1.4 | 4.5 | ± | 1.4 |  |  | 4.5 | ± | 1.4 | 4.6 | ± | 1.4 |  |  | 4.6 | ± | 1.4 | 4.5 | ± | 1.4 |  |
|  | Area4_PK | 4.8 | ± | 1.1 | 4.6 | ± | 1.2 | 5.0 | ± | 1.1 | ¥ |  | 4.9 | ± | 1.2 | 4.8 | ± | 1.2 |  |  | 4.9 | ± | 1.2 | 4.8 | ± | 1.1 |  |
|  | Area4_NE | 5.0 | ± | 1.4 | 4.7 | ± | 1.5 | 4.8 | ± | 1.5 |  |  | 4.7 | ± | 1.6 | 4.9 | ± | 1.4 |  |  | 4.9 | ± | 1.5 | 4.7 | ± | 1.4 |  |
|  | Area5_PK | 4.5 | ± | 1.2 | 4.2 | ± | 1.3 | 4.7 | ± | 1.2 | ¥ |  | 4.7 | ± | 1.1 | 4.3 | ± | 1.3 | * |  | 4.5 | ± | 1.3 | 4.4 | ± | 1.2 |  |
|  | Area5_NE | 5.0 | ± | 1.3 | 4.7 | ± | 1.4 | 4.8 | ± | 1.4 |  |  | 4.7 | ± | 1.4 | 4.9 | ± | 1.3 | * |  | 4.8 | ± | 1.4 | 4.8 | ± | 1.3 |  |
|  | Area6_PK | 4.9 | ± | 1.2 | 4.7 | ± | 1.3 | 5.1 | ± | 1.2 | ¥ |  | 5.2 | ± | 1.1 | 4.7 | ± | 1.3 | * |  | 4.9 | ± | 1.2 | 4.9 | ± | 1.2 |  |
|  | Area6_NE | 5.0 | ± | 1.4 | 4.7 | ± | 1.5 | 4.7 | ± | 1.6 |  |  | 4.6 | ± | 1.6 | 4.9 | ± | 1.4 |  |  | 4.8 | ± | 1.5 | 4.8 | ± | 1.5 |  |
| **Area 1** | PK_1-1 | 4.4 | ± | 1.5 | 4.4 | ± | 1.5 | 5.0 | ± | 1.5 | # ¥ |  | 4.7 | ± | 1.6 | 4.6 | ± | 1.5 |  |  | 4.6 | ± | 1.6 | 4.8 | ± | 1.4 |  |
|  | PK_1-2 | 4.1 | ± | 1.5 | 4.1 | ± | 1.5 | 4.4 | ± | 1.5 | # ¥ |  | 4.3 | ± | 1.6 | 4.2 | ± | 1.5 |  |  | 4.0 | ± | 1.5 | 4.4 | ± | 1.5 | * |
|  | PK_1-3 | 4.0 | ± | 1.6 | 4.1 | ± | 1.6 | 4.6 | ± | 1.5 | # ¥ |  | 4.3 | ± | 1.5 | 4.2 | ± | 1.6 |  |  | 4.1 | ± | 1.6 | 4.4 | ± | 1.5 |  |
|  | PK_1-4 | 4.3 | ± | 1.7 | 4.3 | ± | 1.5 | 4.7 | ± | 1.5 | # ¥ |  | 4.5 | ± | 1.6 | 4.4 | ± | 1.5 |  |  | 4.3 | ± | 1.6 | 4.6 | ± | 1.5 |  |
|  | PK_1-5 | 4.5 | ± | 1.6 | 4.3 | ± | 1.5 | 4.8 | ± | 1.5 | ¥ |  | 4.7 | ± | 1.5 | 4.5 | ± | 1.5 |  |  | 4.5 | ± | 1.6 | 4.7 | ± | 1.4 |  |
|  | PK_1-6 | 4.8 | ± | 1.5 | 4.6 | ± | 1.5 | 5.2 | ± | 1.4 | # ¥ |  | 5.0 | ± | 1.5 | 4.8 | ± | 1.5 |  |  | 4.8 | ± | 1.5 | 4.9 | ± | 1.4 |  |
|  | PK_1-7 | 4.6 | ± | 1.6 | 4.4 | ± | 1.5 | 4.8 | ± | 1.5 | ¥ |  | 4.7 | ± | 1.6 | 4.6 | ± | 1.5 |  |  | 4.5 | ± | 1.6 | 4.8 | ± | 1.4 |  |
|  | NE_1-1 | 5.0 | ± | 1.5 | 4.5 | ± | 1.6 | 4.5 | ± | 1.6 | * # |  | 4.5 | ± | 1.7 | 4.7 | ± | 1.5 |  |  | 4.8 | ± | 1.7 | 4.5 | ± | 1.5 |  |
|  | NE_1-2 | 4.7 | ± | 1.7 | 4.5 | ± | 1.6 | 4.5 | ± | 1.5 |  |  | 4.5 | ± | 1.7 | 4.6 | ± | 1.5 |  |  | 4.7 | ± | 1.6 | 4.4 | ± | 1.5 | * |
|  | NE_1-3 | 4.7 | ± | 1.6 | 4.6 | ± | 1.6 | 4.5 | ± | 1.5 |  |  | 4.5 | ± | 1.7 | 4.6 | ± | 1.5 |  |  | 4.7 | ± | 1.6 | 4.4 | ± | 1.5 |  |
|  | NE_1-4 | 4.9 | ± | 1.6 | 4.5 | ± | 1.6 | 4.6 | ± | 1.6 |  |  | 4.5 | ± | 1.7 | 4.8 | ± | 1.5 |  |  | 4.8 | ± | 1.6 | 4.5 | ± | 1.5 |  |
|  | NE_1-5 | 5.0 | ± | 1.6 | 4.6 | ± | 1.6 | 4.6 | ± | 1.6 |  |  | 4.6 | ± | 1.6 | 4.8 | ± | 1.5 |  |  | 4.8 | ± | 1.6 | 4.5 | ± | 1.5 | * |
|  | NE_1-6 | 5.1 | ± | 1.6 | 4.6 | ± | 1.6 | 4.5 | ± | 1.7 | * # |  | 4.6 | ± | 1.7 | 4.8 | ± | 1.6 |  |  | 4.8 | ± | 1.7 | 4.5 | ± | 1.6 |  |
|  | NE_1-7 | 4.9 | ± | 1.5 | 4.5 | ± | 1.6 | 4.5 | ± | 1.5 |  |  | 4.4 | ± | 1.6 | 4.7 | ± | 1.5 |  |  | 4.8 | ± | 1.6 | 4.4 | ± | 1.6 | * |
| **Area 2** | PK_2-1 | 4.9 | ± | 1.3 | 4.6 | ± | 1.3 | 4.8 | ± | 1.3 |  |  | 4.8 | ± | 1.3 | 4.8 | ± | 1.2 |  |  | 4.8 | ± | 1.3 | 4.7 | ± | 1.2 |  |
|  | PK_2-2 | 3.9 | ± | 1.5 | 3.9 | ± | 1.4 | 4.2 | ± | 1.5 |  |  | 4.1 | ± | 1.5 | 4.0 | ± | 1.5 |  |  | 4.0 | ± | 1.5 | 4.0 | ± | 1.4 |  |
|  | PK_2-3 | 4.2 | ± | 1.6 | 3.9 | ± | 1.5 | 4.2 | ± | 1.4 |  |  | 4.2 | ± | 1.6 | 4.1 | ± | 1.4 |  |  | 4.1 | ± | 1.5 | 4.1 | ± | 1.4 |  |
|  | PK_2-4 | 4.6 | ± | 1.4 | 4.5 | ± | 1.4 | 4.7 | ± | 1.3 |  |  | 4.7 | ± | 1.4 | 4.5 | ± | 1.3 |  |  | 4.6 | ± | 1.4 | 4.6 | ± | 1.3 |  |
|  | NE_2-1 | 5.0 | ± | 1.5 | 4.6 | ± | 1.5 | 4.6 | ± | 1.5 | * # |  | 4.6 | ± | 1.6 | 4.8 | ± | 1.4 |  |  | 4.9 | ± | 1.5 | 4.5 | ± | 1.5 | * |
|  | NE_2-2 | 4.9 | ± | 1.6 | 4.6 | ± | 1.6 | 4.5 | ± | 1.5 |  |  | 4.5 | ± | 1.6 | 4.8 | ± | 1.5 |  |  | 4.9 | ± | 1.6 | 4.5 | ± | 1.6 | * |
|  | NE_2-3 | 4.9 | ± | 1.5 | 4.5 | ± | 1.5 | 4.5 | ± | 1.5 |  |  | 4.5 | ± | 1.6 | 4.7 | ± | 1.5 |  |  | 4.8 | ± | 1.5 | 4.4 | ± | 1.5 | * |
|  | NE_2-4 | 5.1 | ± | 1.5 | 4.5 | ± | 1.6 | 4.6 | ± | 1.5 | * # |  | 4.6 | ± | 1.6 | 4.8 | ± | 1.5 |  |  | 4.9 | ± | 1.5 | 4.5 | ± | 1.6 | * |
| **Area 3** | PK_3-1 | 4.4 | ± | 1.1 | 4.2 | ± | 1.3 | 4.5 | ± | 1.2 |  |  | 4.4 | ± | 1.2 | 4.3 | ± | 1.2 |  |  | 4.3 | ± | 1.3 | 4.4 | ± | 1.2 |  |
|  | PK_3-2 | 4.7 | ± | 1.1 | 4.5 | ± | 1.3 | 4.8 | ± | 1.2 |  |  | 4.7 | ± | 1.2 | 4.6 | ± | 1.2 |  |  | 4.7 | ± | 1.2 | 4.6 | ± | 1.2 |  |
|  | PK_3-3 | 4.7 | ± | 1.1 | 4.5 | ± | 1.3 | 4.8 | ± | 1.2 |  |  | 4.8 | ± | 1.2 | 4.6 | ± | 1.1 | * |  | 4.7 | ± | 1.2 | 4.7 | ± | 1.2 |  |
|  | PK_3-4 | 4.9 | ± | 1.2 | 4.6 | ± | 1.3 | 4.9 | ± | 1.2 |  |  | 4.9 | ± | 1.2 | 4.7 | ± | 1.2 | * |  | 4.7 | ± | 1.3 | 4.9 | ± | 1.1 |  |
|  | PK_3-5 | 5.0 | ± | 1.2 | 4.7 | ± | 1.4 | 5.0 | ± | 1.3 |  |  | 5.1 | ± | 1.3 | 4.8 | ± | 1.3 | * |  | 5.0 | ± | 1.4 | 4.8 | ± | 1.3 |  |
|  | NE_3-1 | 4.6 | ± | 1.5 | 4.4 | ± | 1.4 | 4.4 | ± | 1.4 |  |  | 4.4 | ± | 1.4 | 4.5 | ± | 1.5 |  |  | 4.5 | ± | 1.5 | 4.4 | ± | 1.4 |  |
|  | NE_3-2 | 4.7 | ± | 1.5 | 4.5 | ± | 1.5 | 4.5 | ± | 1.5 |  |  | 4.5 | ± | 1.5 | 4.6 | ± | 1.5 |  |  | 4.6 | ± | 1.5 | 4.5 | ± | 1.5 |  |
|  | NE_3-3 | 4.8 | ± | 1.4 | 4.4 | ± | 1.5 | 4.5 | ± | 1.5 |  |  | 4.5 | ± | 1.5 | 4.6 | ± | 1.5 |  |  | 4.6 | ± | 1.5 | 4.5 | ± | 1.4 |  |
|  | NE_3-4 | 4.8 | ± | 1.5 | 4.5 | ± | 1.5 | 4.5 | ± | 1.5 |  |  | 4.5 | ± | 1.5 | 4.7 | ± | 1.5 |  |  | 4.6 | ± | 1.5 | 4.5 | ± | 1.5 |  |
|  | NE_3-5 | 4.8 | ± | 1.6 | 4.4 | ± | 1.6 | 4.6 | ± | 1.6 |  |  | 4.5 | ± | 1.7 | 4.6 | ± | 1.6 |  |  | 4.6 | ± | 1.6 | 4.5 | ± | 1.6 |  |
| **Area 4** | PK_4-1 | 4.7 | ± | 1.2 | 4.5 | ± | 1.3 | 5.0 | ± | 1.1 |  |  | 4.8 | ± | 1.2 | 4.7 | ± | 1.2 |  |  | 4.8 | ± | 1.2 | 4.8 | ± | 1.2 |  |
|  | PK_4-2 | 4.9 | ± | 1.2 | 4.7 | ± | 1.4 | 5.0 | ± | 1.2 |  |  | 5.0 | ± | 1.2 | 4.8 | ± | 1.3 |  |  | 4.9 | ± | 1.3 | 4.8 | ± | 1.2 |  |
|  | PK_4-3 | 4.7 | ± | 1.2 | 4.7 | ± | 1.3 | 5.0 | ± | 1.2 |  |  | 4.9 | ± | 1.2 | 4.7 | ± | 1.2 |  |  | 4.9 | ± | 1.3 | 4.7 | ± | 1.2 |  |
|  | NE_4-1 | 4.9 | ± | 1.4 | 4.6 | ± | 1.5 | 4.7 | ± | 1.6 |  |  | 4.7 | ± | 1.6 | 4.8 | ± | 1.4 |  |  | 4.8 | ± | 1.5 | 4.6 | ± | 1.5 |  |
|  | NE_4-2 | 5.0 | ± | 1.4 | 4.7 | ± | 1.5 | 4.8 | ± | 1.6 |  |  | 4.7 | ± | 1.6 | 4.9 | ± | 1.4 |  |  | 4.9 | ± | 1.5 | 4.7 | ± | 1.5 |  |
|  | NE_4-3 | 5.0 | ± | 1.4 | 4.7 | ± | 1.5 | 4.7 | ± | 1.6 |  |  | 4.7 | ± | 1.6 | 4.9 | ± | 1.4 |  |  | 4.9 | ± | 1.6 | 4.7 | ± | 1.4 |  |
| **Area 5** | PK_5-1 | 4.4 | ± | 1.4 | 4.1 | ± | 1.6 | 4.7 | ± | 1.4 | ¥ |  | 4.7 | ± | 1.3 | 4.2 | ± | 1.5 | * |  | 4.4 | ± | 1.5 | 4.4 | ± | 1.4 |  |
|  | PK_5-2 | 4.5 | ± | 1.3 | 4.3 | ± | 1.4 | 4.7 | ± | 1.3 | ¥ |  | 4.8 | ± | 1.3 | 4.3 | ± | 1.4 | * |  | 4.5 | ± | 1.4 | 4.4 | ± | 1.3 |  |
|  | PK_5-3 | 4.5 | ± | 1.2 | 4.3 | ± | 1.4 | 4.7 | ± | 1.3 | ¥ |  | 4.8 | ± | 1.2 | 4.3 | ± | 1.3 | * |  | 4.5 | ± | 1.4 | 4.5 | ± | 1.2 |  |
|  | PK_5-4 | 4.3 | ± | 1.4 | 4.1 | ± | 1.4 | 4.6 | ± | 1.3 | ¥ |  | 4.6 | ± | 1.3 | 4.2 | ± | 1.4 | * |  | 4.3 | ± | 1.4 | 4.4 | ± | 1.4 |  |
|  | PK_5-5 | 4.7 | ± | 1.3 | 4.4 | ± | 1.4 | 4.8 | ± | 1.2 | ¥ |  | 4.9 | ± | 1.2 | 4.4 | ± | 1.4 | * |  | 4.7 | ± | 1.4 | 4.6 | ± | 1.3 |  |
|  | PK_5-6 | 4.7 | ± | 1.4 | 4.4 | ± | 1.4 | 4.8 | ± | 1.2 | ¥ |  | 4.8 | ± | 1.2 | 4.5 | ± | 1.4 | * |  | 4.7 | ± | 1.4 | 4.6 | ± | 1.3 |  |
|  | PK_5-7 | 4.2 | ± | 1.5 | 3.9 | ± | 1.5 | 4.4 | ± | 1.4 | ¥ |  | 4.4 | ± | 1.4 | 4.1 | ± | 1.5 | * |  | 4.2 | ± | 1.5 | 4.2 | ± | 1.4 |  |
|  | PK_5-8 | 4.3 | ± | 1.5 | 4.0 | ± | 1.5 | 4.5 | ± | 1.4 | ¥ |  | 4.5 | ± | 1.3 | 4.2 | ± | 1.5 |  |  | 4.3 | ± | 1.4 | 4.2 | ± | 1.4 |  |
|  | NE_5-1 | 5.0 | ± | 1.5 | 4.7 | ± | 1.5 | 4.8 | ± | 1.4 |  |  | 4.7 | ± | 1.5 | 4.9 | ± | 1.4 |  |  | 4.9 | ± | 1.5 | 4.8 | ± | 1.4 |  |
|  | NE_5-2 | 4.9 | ± | 1.4 | 4.7 | ± | 1.4 | 4.8 | ± | 1.4 |  |  | 4.7 | ± | 1.5 | 4.9 | ± | 1.3 | * |  | 4.8 | ± | 1.5 | 4.8 | ± | 1.4 |  |
|  | NE_5-3 | 4.9 | ± | 1.4 | 4.7 | ± | 1.4 | 4.7 | ± | 1.4 |  |  | 4.6 | ± | 1.5 | 4.9 | ± | 1.4 | * |  | 4.8 | ± | 1.4 | 4.7 | ± | 1.4 |  |
|  | NE_5-4 | 4.9 | ± | 1.5 | 4.5 | ± | 1.5 | 4.7 | ± | 1.5 |  |  | 4.5 | ± | 1.6 | 4.8 | ± | 1.5 | * |  | 4.7 | ± | 1.6 | 4.7 | ± | 1.4 |  |
|  | NE_5-5 | 5.0 | ± | 1.4 | 4.6 | ± | 1.5 | 4.8 | ± | 1.5 |  |  | 4.6 | ± | 1.6 | 4.9 | ± | 1.4 |  |  | 4.8 | ± | 1.5 | 4.8 | ± | 1.4 |  |
|  | NE_5-6 | 5.0 | ± | 1.4 | 4.7 | ± | 1.5 | 4.8 | ± | 1.4 |  |  | 4.7 | ± | 1.5 | 4.9 | ± | 1.4 |  |  | 4.9 | ± | 1.5 | 4.8 | ± | 1.4 |  |
|  | NE_5-7 | 5.1 | ± | 1.5 | 4.7 | ± | 1.7 | 4.8 | ± | 1.5 | * |  | 4.7 | ± | 1.6 | 5.0 | ± | 1.5 |  |  | 4.8 | ± | 1.6 | 4.9 | ± | 1.5 |  |
|  | NE_5-8 | 5.2 | ± | 1.4 | 4.7 | ± | 1.7 | 4.9 | ± | 1.5 | * |  | 4.8 | ± | 1.6 | 5.0 | ± | 1.5 |  |  | 4.9 | ± | 1.6 | 4.9 | ± | 1.5 |  |
| **Area 6** | PK_6-1 | 4.5 | ± | 1.6 | 4.5 | ± | 1.5 | 5.0 | ± | 1.4 | * # |  | 5.1 | ± | 1.3 | 4.4 | ± | 1.6 | * |  | 4.6 | ± | 1.6 | 4.8 | ± | 1.4 |  |
|  | PK_6-2 | 5.0 | ± | 1.3 | 4.7 | ± | 1.4 | 5.1 | ± | 1.3 | ¥ |  | 5.2 | ± | 1.2 | 4.8 | ± | 1.3 | * |  | 4.9 | ± | 1.3 | 5.0 | ± | 1.3 |  |
|  | PK_6-3 | 5.1 | ± | 1.2 | 4.8 | ± | 1.4 | 5.2 | ± | 1.3 | ¥ |  | 5.3 | ± | 1.2 | 4.9 | ± | 1.3 | * |  | 5.1 | ± | 1.3 | 5.1 | ± | 1.3 |  |
|  | PK_6-4 | 4.9 | ± | 1.3 | 4.7 | ± | 1.4 | 5.1 | ± | 1.2 | ¥ |  | 5.2 | ± | 1.2 | 4.7 | ± | 1.4 | * |  | 4.9 | ± | 1.3 | 5.0 | ± | 1.3 |  |
|  | PK_6-5 | 4.8 | ± | 1.3 | 4.7 | ± | 1.4 | 5.1 | ± | 1.2 | ¥ |  | 5.1 | ± | 1.2 | 4.7 | ± | 1.4 | * |  | 4.9 | ± | 1.3 | 4.9 | ± | 1.3 |  |
|  | PK_6-6 | 4.9 | ± | 1.4 | 4.7 | ± | 1.4 | 5.1 | ± | 1.3 | ¥ |  | 5.1 | ± | 1.3 | 4.8 | ± | 1.4 | * |  | 4.9 | ± | 1.4 | 4.9 | ± | 1.4 |  |
|  | PK_6-7 | 4.8 | ± | 1.3 | 4.7 | ± | 1.3 | 5.1 | ± | 1.3 | ¥ |  | 5.1 | ± | 1.2 | 4.7 | ± | 1.3 | * |  | 4.9 | ± | 1.3 | 4.9 | ± | 1.3 |  |
|  | PK_6-8 | 4.8 | ± | 1.4 | 4.6 | ± | 1.4 | 5.0 | ± | 1.3 | ¥ |  | 5.1 | ± | 1.3 | 4.6 | ± | 1.4 | * |  | 4.8 | ± | 1.4 | 4.8 | ± | 1.4 |  |
|  | NE_6-1 | 5.1 | ± | 1.5 | 4.8 | ± | 1.5 | 4.7 | ± | 1.7 |  |  | 4.7 | ± | 1.7 | 4.9 | ± | 1.5 |  |  | 4.9 | ± | 1.7 | 4.8 | ± | 1.5 |  |
|  | NE_6-2 | 4.9 | ± | 1.5 | 4.7 | ± | 1.5 | 4.6 | ± | 1.6 |  |  | 4.6 | ± | 1.6 | 4.8 | ± | 1.5 |  |  | 4.8 | ± | 1.6 | 4.7 | ± | 1.5 |  |
|  | NE_6-3 | 4.9 | ± | 1.5 | 4.7 | ± | 1.5 | 4.6 | ± | 1.6 |  |  | 4.5 | ± | 1.7 | 4.8 | ± | 1.5 |  |  | 4.7 | ± | 1.5 | 4.7 | ± | 1.6 |  |
|  | NE_6-4 | 4.9 | ± | 1.5 | 4.7 | ± | 1.5 | 4.6 | ± | 1.6 |  |  | 4.6 | ± | 1.7 | 4.8 | ± | 1.4 |  |  | 4.7 | ± | 1.5 | 4.7 | ± | 1.5 |  |
|  | NE_6-5 | 5.0 | ± | 1.5 | 4.8 | ± | 1.5 | 4.7 | ± | 1.6 |  |  | 4.6 | ± | 1.7 | 5.0 | ± | 1.4 |  |  | 4.9 | ± | 1.5 | 4.8 | ± | 1.5 |  |
|  | NE_6-6 | 5.1 | ± | 1.5 | 4.7 | ± | 1.6 | 4.7 | ± | 1.7 | * |  | 4.6 | ± | 1.7 | 5.0 | ± | 1.5 |  |  | 4.9 | ± | 1.6 | 4.8 | ± | 1.6 |  |
|  | NE_6-7 | 5.0 | ± | 1.5 | 4.7 | ± | 1.6 | 4.7 | ± | 1.6 |  |  | 4.6 | ± | 1.7 | 4.9 | ± | 1.5 |  |  | 4.8 | ± | 1.6 | 4.8 | ± | 1.5 |  |
|  | NE_6-8 | 5.1 | ± | 1.5 | 4.8 | ± | 1.5 | 4.7 | ± | 1.6 |  |  | 4.6 | ± | 1.7 | 5.0 | ± | 1.5 |  |  | 4.8 | ± | 1.6 | 4.8 | ± | 1.5 |  |
|  |  | * (<10 years vs 10-20 years); # (<10 years vs ≥21 years); ¥ (10-20 years vs ≥21 years) | | | | | | | | | |  |  |  |  |  |  |  |  |  |  |  |  |  |  |  |  |
|  |  |  |  |  |  |  |  |  |  |  |  |  |  |  |  |  |  |  |  |  |  |  |  |  |  |  |  |
