# Supplementary material for: Judo for older adults: the coaches' knowledge and needs of education
Source: Front Sports Act Living. 2024 Apr 2;6:1375814. doi: 10.3389/fspor.2024.1375814 (PMC11018922; doi:10.3389/fspor.2024.1375814)
Supplement: Supplementary file 3 [file Datasheet3.docx]

Supplementary Material 3. Correlation matrixes regarding Knowledge and Need of Education in relation to individual items.

# Significant correlations are displayed (p≤0.01). PK= Knowledge; NE= Need of Education. Duplicate of data: *

| **Variables** |  | **PK_1-1** | **PK_1-3** | **PK_1-4** | **PK_1-5** | **PK_1-6** | **NE_1-1** | **NE_1-2** | **NE_1-3** | **NE_1-4** | **NE_1-5** | **NE_1-6** |
| --- | --- | --- | --- | --- | --- | --- | --- | --- | --- | --- | --- | --- |
| **PK_1-2** | CC | .743^**^ | .888^**^ | .814^**^ | .722^**^ |  |  |  |  |  |  |  |
|  | p | .000 | .000 | .000 | .000 |  |  |  |  |  |  |  |
| **PK_1-3** | CC | .754^**^ |  | * | * |  |  |  |  |  |  |  |
|  | p | .000 |  |  |  |  |  |  |  |  |  |  |
| **PK_1-4** | CC | .805^**^ | .838^**^ |  | * | * |  |  |  |  |  |  |
|  | p | .000 | .000 |  |  |  |  |  |  |  |  |  |
| **PK_1-5** | CC | .813^**^ | .769^**^ | .859^**^ |  | * |  |  |  |  |  |  |
|  | p | .000 | .000 | .000 |  |  |  |  |  |  |  |  |
| **PK_1-6** | CC | .851^**^ |  | .780^**^ | .853^**^ |  |  |  |  |  |  |  |
|  | p | .000 |  | .000 | .000 |  |  |  |  |  |  |  |
| **PK_1-7** | CC | .755^**^ |  | .764^**^ | .791^**^ | .794^**^ |  |  |  |  |  |  |
|  | p | .000 |  | .000 | .000 | .000 |  |  |  |  |  |  |
| **NE_1-2** | CC |  |  |  |  |  | .773^**^ |  |  |  |  |  |
|  | p |  |  |  |  |  | .000 |  |  |  |  |  |
| **NE_1-3** | CC |  |  |  |  |  | .755^**^ | .941^**^ |  |  |  |  |
|  | p |  |  |  |  |  | .000 | .000 |  |  |  |  |
| **NE_1-4** | CC |  |  |  |  |  | .819^**^ | .846^**^ | .846^**^ |  |  |  |
|  | p |  |  |  |  |  | .000 | .000 | .000 |  |  |  |
| **NE_1-5** | CC |  |  |  |  |  | .867^**^ | .779^**^ | .797^**^ | .889^**^ |  |  |
|  | p |  |  |  |  |  | .000 | .000 | .000 | .000 |  |  |
| **NE_1-6** | CC |  |  |  |  |  | .871^**^ |  | .709^**^ | .808^**^ | .875^**^ |  |
|  | p |  |  |  |  |  | .000 |  | .000 | .000 | .000 |  |
| **NE_1-7** | CC |  |  |  |  |  | .805^**^ | .802^**^ | .795^**^ | .841^**^ | .832^**^ | .830^**^ |
|  | p |  |  |  |  |  | .000 | .000 | .000 | .000 | .000 | .000 |

| **Correlations - PK and NE of individual items of Area 2** | | | | | | | | |
| --- | --- | --- | --- | --- | --- | --- | --- | --- |
| **Variables** |  | **PK_2-1** | **PK_2-2** | **PK_2-3** | **PK_2-4** | **NE_2-1** | **NE_2-2** | **NE_2-3** |
| **PK_2-2** | CC |  |  | * | .713^**^ |  |  |  |
|  | p |  |  |  | .000 |  |  |  |
| **PK_2-3** | CC |  | .816^**^ |  | .757^**^ |  |  |  |
|  | p |  | .000 |  | .000 |  |  |  |
| **NE_2-2** | CC |  |  |  |  | .749^**^ |  | .* |
|  | p |  |  |  |  | .000 |  |  |
| **NE_2-3** | CC |  |  |  |  | .729^**^ | .871^**^ |  |
|  | p |  |  |  |  | .000 | .000 |  |
| **NE_2-4** | CC |  |  |  |  | .818^**^ | .817^**^ | .837^**^ |
|  | p |  |  |  |  | .000 | .000 | .000 |

| **Variables** |  | **PK_**  **3-1** | **PK_**  **3-3** | **PK_**  **3-4** | **PK_**  **3-5** | **NE_**  **3-1** | **NE_**  **3-2** | **NE_**  **3-3** | **NE_**  **3-4** |
| --- | --- | --- | --- | --- | --- | --- | --- | --- | --- |
| **PK_3-2** | CC |  | .861^**^ | .796^**^ |  |  |  |  |  |
|  | p |  | .000 | .000 |  |  |  |  |  |
| **PK_3-3** | CC |  |  | .833^**^ | .732^**^ |  |  |  |  |
|  | p |  |  | .000 | .000 |  |  |  |  |
| **PK_3-4** | CC |  | .833^**^ |  | .780^**^ |  |  |  |  |
|  | p |  | .000 |  | .000 |  |  |  |  |
| **NE_3-2** | CC |  |  |  |  | .826^**^ |  | .903^**^ | .909^**^ |
|  | p |  |  |  |  | .000 |  | .000 | .000 |
| **NE_3-3** | CC |  |  |  |  | .818^**^ | .903^**^ |  | .914^**^ |
|  | p |  |  |  |  | .000 | .000 |  | .000 |
| **NE_3-4** | CC |  |  |  |  | .779^**^ | .909^**^ | .914^**^ |  |
|  | p |  |  |  |  | .000 | .000 | .000 |  |
| **NE_3-5** | CC |  |  |  |  | .740^**^ | .846^**^ | .885^**^ | .884^**^ |
|  | p |  |  |  |  | .000 | .000 | .000 | .000 |

| **Variables** |  | **PK_4-1** | **PK_4-3** | **NE_4-1** | **NE_4-2** |
| --- | --- | --- | --- | --- | --- |
| **PK_4-2** | CC | .836^**^ | .856^**^ |  |  |
|  | p | .000 | .000 |  |  |
| **PK_4-3** | CC | .796^**^ |  |  |  |
|  | p | .000 |  |  |  |
| **NE_4-2** | CC |  |  | .921^**^ |  |
|  | p |  |  | .000 |  |
| **NE_4-3** | CC |  |  | .901^**^ | .934^**^ |
|  | p |  |  | .000 | .000 |

| **Variables** |  | **PK_5-1** | **PK_5-3** | **PK_5-4** | **PK_5-5** | **PK_5-6** | **PK_5-7** | **PK_5-8** | **NE_5-1** | **NE_5-2** | **NE_5-3** | **NE_5-4** | **NE_5-5** | **NE_5-6** | **NE_5-7** |
| --- | --- | --- | --- | --- | --- | --- | --- | --- | --- | --- | --- | --- | --- | --- | --- |
| **PK_5-2** | CC | .858^**^ | .869^**^ | .721^**^ | .799^**^ | .793^**^ |  | .700^**^ |  |  |  |  |  |  |  |
|  | p | .000 | .000 | .000 | .000 | .000 |  | .000 |  |  |  |  |  |  |  |
| **PK_5-3** | CC | .788^**^ |  | * | * | * |  |  |  |  |  |  |  |  |  |
|  | p | .000 |  |  |  |  |  |  |  |  |  |  |  |  |  |
| **PK_5-4** | CC |  | .773^**^ |  | * | * | * |  |  |  |  |  |  |  |  |
|  | p |  | .000 |  |  |  |  |  |  |  |  |  |  |  |  |
| **PK_5-5** | CC | .743^**^ | .814^**^ | .776^**^ |  | * |  | .717^**^ |  |  |  |  |  |  |  |
|  | p | .000 | .000 | .000 |  |  |  | .000 |  |  |  |  |  |  |  |
| **PK_5-6** | CC | .739^**^ | .805^**^ | .730^**^ | .869^**^ |  | * | .762^**^ |  |  |  |  |  |  |  |
|  | p | .000 | .000 | .000 | .000 |  |  | .000 |  |  |  |  |  |  |  |
| **PK_5-7** | CC |  |  | .704^**^ |  | .701^**^ |  | .868^**^ |  |  |  |  |  |  |  |
|  | p |  |  | .000 |  | .000 |  | .000 |  |  |  |  |  |  |  |
| **NE_5-2** | CC |  |  |  |  |  |  |  | .923^**^ |  |  |  |  |  |  |
|  | p |  |  |  |  |  |  |  | .000 |  |  |  |  |  |  |
| **NE_5-3** | CC |  |  |  |  |  |  |  | .902^**^ | .908^**^ |  |  |  |  |  |
|  | p |  |  |  |  |  |  |  | .000 | .000 |  |  |  |  |  |
| **NE_5-4** | CC |  |  |  |  |  |  |  | .827^**^ | .835^**^ | .838^**^ |  |  |  |  |
|  | p |  |  |  |  |  |  |  | .000 | .000 | .000 |  |  |  |  |
| **NE_5-5** | CC |  |  |  |  |  |  |  | .898^**^ | .869^**^ | .883^**^ | .860^**^ |  |  |  |
|  | p |  |  |  |  |  |  |  | .000 | .000 | .000 | .000 |  |  |  |
| **NE_5-6** | CC |  |  |  |  |  |  |  | .902^**^ | .887^**^ | .881^**^ | .853^**^ | .925^**^ |  |  |
|  | p |  |  |  |  |  |  |  | .000 | .000 | .000 | .000 | .000 |  |  |
| **NE_5-7** | CC |  |  |  |  |  |  |  | .749^**^ | .751^**^ | .749^**^ | .832^**^ | .795^**^ | .815^**^ |  |
|  | p |  |  |  |  |  |  |  | .000 | .000 | .000 | .000 | .000 | .000 |  |
| **NE_5-8** | CC |  |  |  |  |  |  |  | .789^**^ | .802^**^ | .776^**^ | .837^**^ | .837^**^ | .852^**^ | .940^**^ |
|  | p |  |  |  |  |  |  |  | .000 | .000 | .000 | .000 | .000 | .000 | .000 |

| **Correlations - PK and NE of individual items of Area 6** | | | | | | | | | | | | | | | |
| --- | --- | --- | --- | --- | --- | --- | --- | --- | --- | --- | --- | --- | --- | --- | --- |
| **Variables** |  | **PK_6-1** | **PK_6-2** | **PK_6-3** | **PK_6-4** | **PK_6-5** | **PK_6-6** | **PK_6-7** | **NE_6-1** | **NE_6-2** | **NE_6-3** | **NE_6-4** | **NE_6-5** | **NE_6-6** | **NE_6-7** |
| **PK_6-2** | CC | .803^**^ |  |  |  |  |  |  |  |  |  |  |  |  |  |
|  | p | .000 |  |  |  |  |  |  |  |  |  |  |  |  |  |
| **PK_6-3** | CC | .759^**^ | .879^**^ |  |  |  |  |  |  |  |  |  |  |  |  |
|  | p | .000 | .000 |  |  |  |  |  |  |  |  |  |  |  |  |
| **PK_6-4** | CC | .781^**^ | .812^**^ | .859^**^ |  |  |  |  |  |  |  |  |  |  |  |
|  | p | .000 | .000 | .000 |  |  |  |  |  |  |  |  |  |  |  |
| **PK_6-5** | CC | .757^**^ | .809^**^ | .815^**^ | .871^**^ |  |  |  |  |  |  |  |  |  |  |
|  | p | .000 | .000 | .000 | .000 |  |  |  |  |  |  |  |  |  |  |
| **PK_6-6** | CC | .711^**^ | .760^**^ | .794^**^ | .787^**^ | .809^**^ |  |  |  |  |  |  |  |  |  |
|  | p | .000 | .000 | .000 | .000 | .000 |  |  |  |  |  |  |  |  |  |
| **PK_6-7** | CC | .749^**^ | .775^**^ | .816^**^ | .802^**^ | .838^**^ | .885^**^ |  |  |  |  |  |  |  |  |
|  | p | .000 | .000 | .000 | .000 | .000 | .000 |  |  |  |  |  |  |  |  |
| **PK_6-8** | CC | .703^**^ | .704^**^ | .756^**^ | .788^**^ | .797^**^ | .836^**^ | .859^**^ |  |  |  |  |  |  |  |
|  | p | .000 | .000 | .000 | .000 | .000 | .000 | .000 |  |  |  |  |  |  |  |
| **NE_6-2** | CC |  |  |  |  |  |  |  | .904^**^ |  |  |  |  |  |  |
|  | p |  |  |  |  |  |  |  | .000 |  |  |  |  |  |  |
| **NE_6-3** | CC |  |  |  |  |  |  |  | .896^**^ | .950^**^ |  |  |  |  |  |
|  | p |  |  |  |  |  |  |  | .000 | .000 |  |  |  |  |  |
| **NE_6-4** | CC |  |  |  |  |  |  |  | .869^**^ | .915^**^ | .932^**^ |  |  |  |  |
|  | p |  |  |  |  |  |  |  | .000 | .000 | .000 |  |  |  |  |
| **NE_6-5** | CC |  |  |  |  |  |  |  | .885^**^ | .918^**^ | .915^**^ | .929^**^ |  |  |  |
|  | p |  |  |  |  |  |  |  | .000 | .000 | .000 | .000 |  |  |  |
| **NE_6-6** | CC |  |  |  |  |  |  |  | .846^**^ | .888^**^ | .888^**^ | .889^**^ | .912^**^ |  |  |
|  | p |  |  |  |  |  |  |  | .000 | .000 | .000 | .000 | .000 |  |  |
| **NE_6-7** | CC |  |  |  |  |  |  |  | .848^**^ | .902^**^ | .920^**^ | .910^**^ | .921^**^ | .928^**^ |  |
|  | p |  |  |  |  |  |  |  | .000 | .000 | .000 | .000 | .000 | .000 |  |
| **NE_6-8** | CC |  |  |  |  |  |  |  | .834^**^ | .876^**^ | .883^**^ | .894^**^ | .891^**^ | .918^**^ | .938^**^ |
|  | p |  |  |  |  |  |  |  | .000 | .000 | .000 | .000 | .000 | .000 | .000 |

# 
