# Supplementary material for: Judo for older adults: the coaches' knowledge and needs of education
Source: Front Sports Act Living. 2024 Apr 2;6:1375814. doi: 10.3389/fspor.2024.1375814 (PMC11018922; doi:10.3389/fspor.2024.1375814)
Supplement: Supplementary file 4 [file Datasheet4.docx]

***Supplementary Material 4.***

**Figure 1 and 2 -** Graphical representation summary of the main effects (p<0.05) emerged in relation to individual items and the considered independent variables within the PK and NE domains (see also Supplementary Material 2).

**Figure 1. Need for education.**


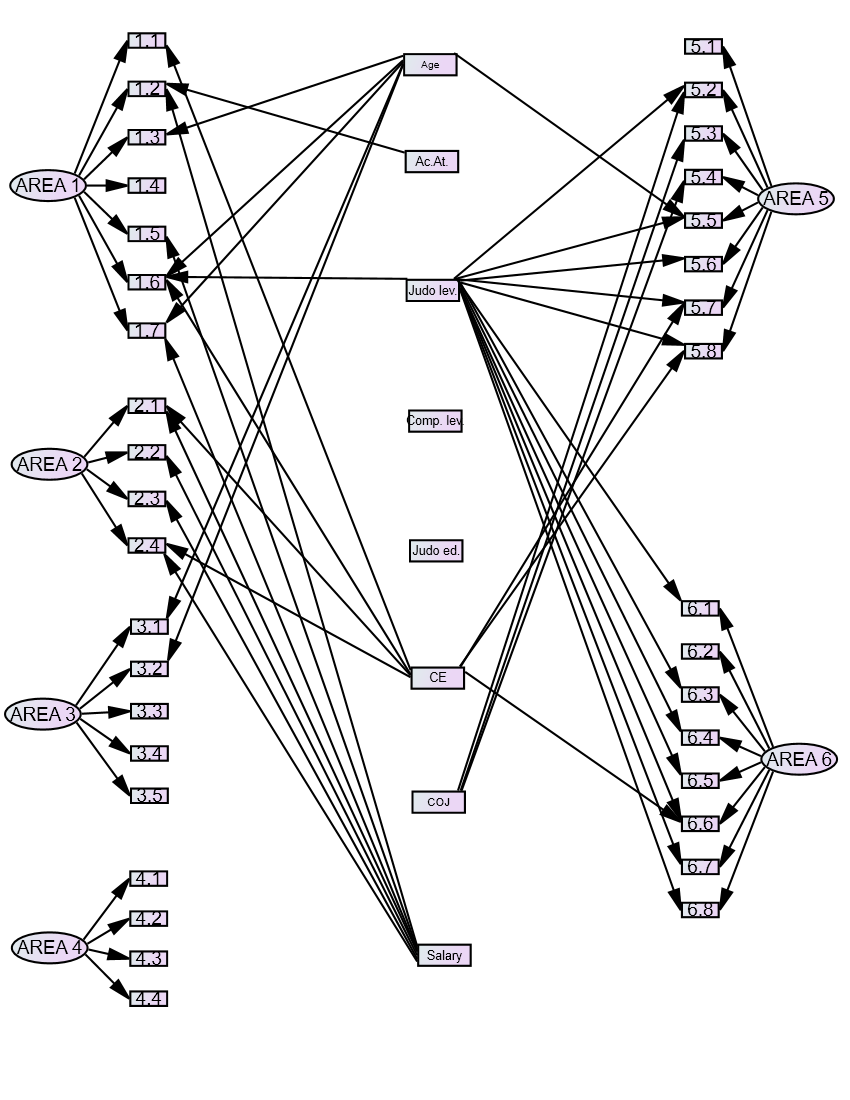


Note: **Ac. At.** = highest academic attainment; **Judo lev.** = judo level; **Comp. lev.** = former competition level; **Judo ed.** = judo education level; **CE** = coaching experience; **COJ** = coaching older judo practitioners; **Salary** = salary for judo. Lines with arrow identify a statistical significance (p>0.05).

**Figure 2. Perceived knowledge.**


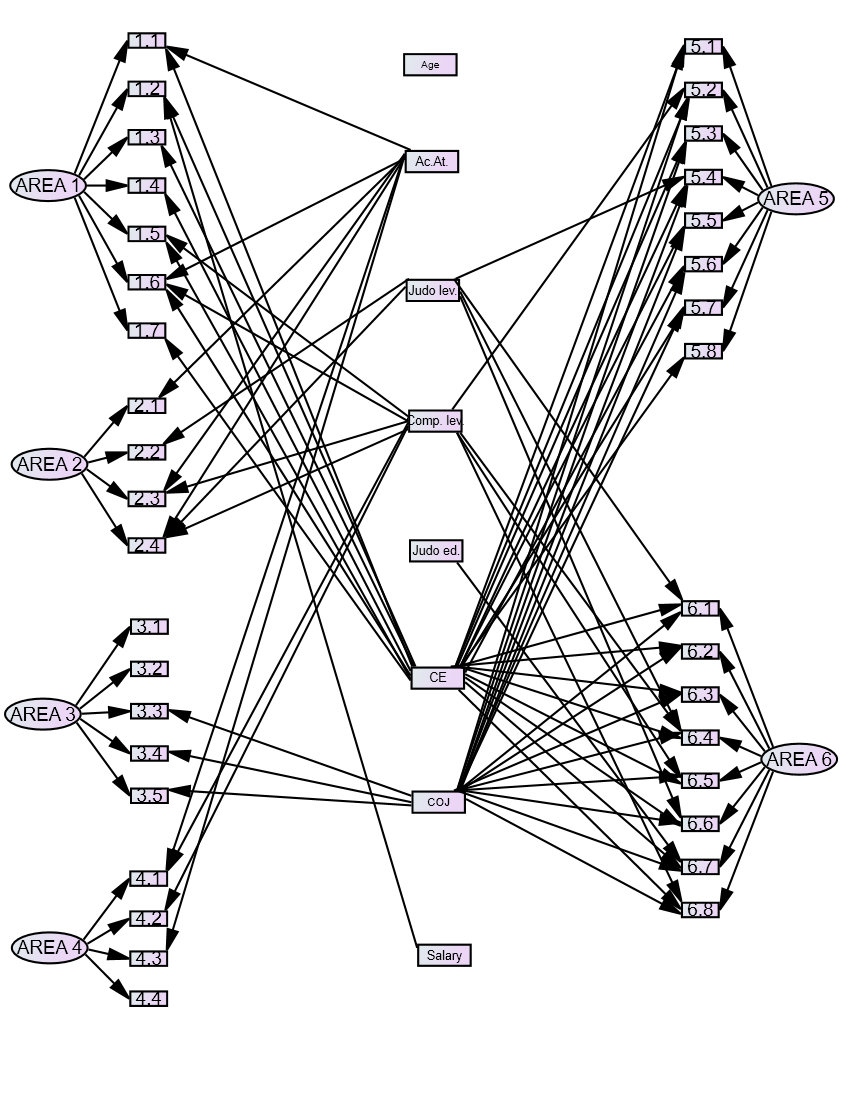


Note: **Ac. At.** = highest academic attainment; **Judo lev.** = judo level; **Comp. lev.** = former competition level; **Judo ed.** = judo education level; **CE** = coaching experience; **COJ** = coaching older judo practitioners; **Salary** = salary for judo. Lines with arrow identify a statistical significance (p>0.05).
